# Is psychosis a multisystem disorder? A meta-review of central nervous system, immune, cardiometabolic, and endocrine alterations in first-episode psychosis and perspective on potential models

**DOI:** 10.1038/s41380-018-0058-9

**Published:** 2018-05-09

**Authors:** Toby Pillinger, Enrico D’Ambrosio, Robert McCutcheon, Oliver D. Howes

**Affiliations:** 1 IoPPN, King’s College London, De Crespigny Park, London, SE5 8AF UK; 2 MRC London Institute of Medical Sciences (LMS), Du Cane Road, London, W12 0NN UK; 3 Institute of Clinical Sciences (ICS), Faculty of Medicine, Imperial College London, Du Cane Road, London, W12 0NN UK

**Keywords:** Diagnostic markers, Physiology, Schizophrenia

## Abstract

People with psychotic disorders show abnormalities in several organ systems in addition to the central nervous system (CNS); and this contributes to excess mortality. However, it is unclear how strong the evidence is for alterations in non-CNS systems at the onset of psychosis, how the alterations in non-CNS systems compare to those in the CNS, or how they relate to symptoms. Here, we consider these questions, and suggest potential models to account for findings. We conducted a systematic meta-review to summarize effect sizes for both CNS (focusing on brain structural, neurophysiological, and neurochemical parameters) and non-CNS dysfunction (focusing on immune, cardiometabolic, and hypothalamic–pituitary–adrenal (HPA) systems) in first-episode psychosis (FEP). Relevant meta-analyses were identified in a systematic search of Pubmed and the methodological quality of these was assessed using the AMSTAR checklist (A Measurement Tool to Assess Systematic Reviews). Case–control data were extracted from studies included in these meta-analyses. Random effects meta-analyses were re-run and effect size magnitudes for individual parameters were calculated, as were summary effect sizes for each CNS and non-CNS system. We also grouped studies to obtain overall effect sizes for non-CNS and CNS alterations. Robustness of data for non-CNS and CNS parameters was assessed using Rosenthal’s fail-safe N. We next statistically compared summary effect size for overall CNSand overall non-CNS alterations, as well as for each organ system separately. We also examined how non-CNS alterations correlate CNS alterations, and with psychopathological symptoms. Case-control data were extracted for 165 studies comprising a total sample size of 13,440. For people with first episode psychosis compared with healthy controls, we observed alterations in immune parameters (summary effect size: *g* = 1.19), cardiometabolic parameters (*g* = 0.23); HPA parameters (*g* = 0.68); brain structure (*g* = 0.40); neurophysiology (*g* = 0.80); and neurochemistry (*g* = 0.43). Grouping non-CNS organ systems together provided an effect size for overall non-CNS alterations in patients compared with controls (*g* = 0.58), which was not significantly different from the overall CNS alterations effect size (*g* = 0.50). However, the summary effect size for immune alterations was significantly greater than that for brain structural (*P* < 0.001) and neurochemical alterations (*P* < 0.001), while the summary effect size for cardiometabolic alterations was significantly lower than neurochemical (*P* = 0.04), neurophysiological (*P* < 0.001), and brain structural alterations (*P* = 0.001). The summary effect size for HPA alterations was not significantly different from brain structural (*P* = 0.14), neurophysiological (*P* = 0.54), or neurochemical alterations (*P* = 0.22). These outcomes remained similar in antipsychotic naive sensitivity analyses. We found some, but limited and inconsistent, evidence that non-CNS alterations were associated with CNS changes and symptoms in first episode psychosis. Our findings indicate that there are robust alterations in non-CNS systems in psychosis, and that these are broadly similar in magnitude to a range of CNS alterations. We consider models that could account for these findings and discuss implications for future research and treatment.

## Introduction

Schizophrenia and related psychotic disorders have a worldwide lifetime prevalence of ~1% [1]. They are highly disabling conditions with economic costs over $60 billion per year in the United States [2, 3]. Epidemiological studies have established that people with psychotic disorders die 15–20 years earlier than the general population, and that 60% or more of this premature mortality relates to non-CNS, predominantly cardiovascular, causes [4–8]. Poor physical health has traditionally been blamed on the secondary effects of the illness, be that a consequence of the illness itself (e.g., negative symptoms leading to sedentary lifestyle and poor diet) [9], or a consequence of treatment (e.g., second-generation antipsychotic use) [10]. In recent years however, studies in first-episode patients have shown dysfunction in cardiometabolic [11–16], immune [17–21], and hypothalamic pituitary adrenal (HPA) [22–25] systems. This suggests that psychotic disorders involve multiple systems at onset. However, it is unclear how strong the evidence is for abnormalities across these systems, how alterations compare with CNS abnormalities seen in the disorder, or how they relate to symptoms. To address these questions, we perform a meta-review of the magnitude, consistency, and robustness of dysfunction across these systems as assessed using peripheral markers, and compare findings with representative CNS abnormalities in psychosis. We then review the potential models that could explain the associations, before considering both the research and clinical implications of our findings.

## Methods

### Systematic meta-review summarizing effect size magnitudes for central nervous system and non-central nervous system alterations in first-episode psychosis

Full details of methods employed are documented in Supplementary Information (eAppendix 1). Two systematic reviews of meta-analyses were performed according to preferred reporting items for systematic reviews and meta-analyses (PRISMA) guidelines [26] (Supplementary Information, eTable 1). Two reviewers (TP and ED) independently searched Pubmed from 1990 to May week 2 2017 for each systematic meta-review. For the meta-review focussing on non-CNS dysfunction, we focused on meta-analyses of findings in three organ systems established as showing dysregulation in schizophrenia: the immune [27], cardiometabolic [28], and HPA [29] systems. We selected meta-analyses reporting markers of immune, cardiometabolic, and HPA system differences between patient and control groups, rather than differences in rates of diagnoses of conditions based on pre-defined diagnostic criteria [30–33] (e.g., rates of diagnoses of type 2 diabetes mellitus or hypercholesterolemia). The rationale behind this methodology was threefold. First, patients with psychotic illness are less likely to seek medical attention and so there is the risk of under-reporting of diagnoses [34]. Second, certain conditions, such as glucose and lipid dysregulation, develop on a continuum and take time for serum/plasma markers to reach threshold for a diagnosis. For example, changes in glucose regulation occur 4–7 years prior to diagnosis of type 2 diabetes mellitus [35]. Third, physiological alterations that do not meet diagnostic thresholds can, nevertheless, be associated with worsened mortality/morbidity outcomes. For example, there is robust evidence that low-grade inflammation is an independent risk factor for atherosclerosis and cardiovascular disease [36]. For the meta-review focussing on CNS dysfunction, we focused on meta-analyses of parameters previously identified in an expert review as key neurobiological alterations seen in schizophrenia [37], covering alterations in brain structure, neurophysiology, and neurochemistry. The search was limited to first-episode psychosis (FEP) to limit secondary effects of illness.

Patient and control data from the studies referenced in the meta-analyses that our search terms had identified were extracted and all meta-analyses repeated. Data were only extracted for those CNS and non-CNS parameters, where there were significant differences demonstrated between FEP and controls in the original meta-analyses. A two-tailed *P* < 0.05 was deemed significant. A minimum of three studies was required to run a meta-analysis. For non-CNS parameters, random-effects meta-analyses were performed examining immune (interleukin-1β (IL-1β), soluble interleukin-2 receptor (sIL-2R), interleukin-6 (IL-6), tumour necrosis factor-α (TNF-α), transforming growth factor-β (TGF-β), C-reactive protein (CRP), and total lymphocyte count), cardiometabolic (fasting glucose, glucose post-oral glucose tolerance test (OGTT), fasting insulin, insulin resistance (HOMA-IR), total cholesterol, low density lipoprotein (LDL) cholesterol, and triglycerides), and HPA parameters (cortisol awakening response and prolactin concentrations). For CNS parameters, random-effects meta-analyses were performed examining brain structural (total brain volume, total grey matter volume, total CSF volume, right and left hippocampus, right and left lateral ventricle, total thalamus, right and left caudate nucleus), neurophysiological (auditory P300 amplitude and latency, duration deviant mismatch negativity), and neurochemical parameters (frontal, temporal and thalamus *N*-acetylaspartic acid (NAA) levels). A random-effects model was used in all analyses owing to an expectation of heterogeneity of data across studies. Standardized mean differences between patient and control cohort parameters were used as the effect size, determined using Hedges adjusted *g*. The 95% CI of the effect size was also calculated. A criticism of meta-analysis is that it may be based on a biased sample of studies, potentially inflating effect sizes. Thus, for each meta-analysis, Rosenthal’s “fail-safe N” [38] was used to calculate the number of additional null studies (i.e., studies where the effect size is zero) that would be required to increase the *P* value for a given meta-analysis to greater than 0.05. This provides an assessment of how bias could influence the results of a meta-analysis: the greater the number of estimated studies required for the finding to be no longer significant, the less likely the results are secondary to publication or small sample bias and therefore the more robust the finding. Methodological quality of each meta-analysis was assessed using the AMSTAR checklist (A Measurement Tool to Assess Systematic Reviews) (eAppendix 2). Heterogeneity scores for samples within each meta-analysis were also recorded, as assessed using the *χ*^2^ test, *Q*, or *I*^2^ statistic (Tables 1 and 2).

**Table 1:** Meta-analyses examining cardiometabolic, hypothalamic–pituitary–adrenal (HPA) axis, and immune alterations in first-episode psychosis that met inclusion criteria

Meta-analysis	Objective	No. of studies	Study range	Anti-psychotic status	Patient/HC no.	Non-CNS parameter	Effect size	Hetero-geneity (*I*^2^)	AMSTAR
Pillinger et al. [11]	Cardiometabolic profile	16	2003–2016	Minimal (<2 weeks, antipsychotic naive subgroup)	731/614	Fasting glucose	↑ 0.30 *P* = 0.002	58–82%	10/11
						Glucose post-OGTT	↑ 0.61 *P* = 0.007		
						Fasting insulin	↑ 0.47 *P* = 0.04		
						Insulin resistance	↑ 0.44 *P* < 0.001		
						HbA1c	↔ −0.08 *P* > 0.05		
Perry et al. [12]	Cardiometabolic profile	12	2003–2015	Minimal (<1 week)	564/573	Fasting glucose;	↔ 0.06 *P* > 0.05	0–94%	10/11
						Glucose post-OGTT	↑ 0.82 *P* < 0.0001		
						Insulin resistance	↑ 0.26 *P* < 0.0001		
Greenhalgh et al. [13]	Cardiometabolic profile	19	2003–2016	Minimal (<1 week)	911/870	Fasting glucose	↑ 0.21 *P* < 0.001	55–83%	9/11
						Glucose post-OGTT	↑ 0.58 *P* < 0.001		
						Fasting insulin	↑ 0.28 *P* < 0.001		
						Insulin resistance	↑ 0.30 *P* < 0.001		
Misiak et al. [14]	Cardiometabolic profile	19	2000–2016	Antipsychotic naive	866/937	Total cholesterol	↓ −0.16 *P* = 0.003	0–74%	11/11
						HDL cholesterol	↓ −0.27 *P* = 0.018		
						LDL cholesterol	↓ −0.13 *P* = 0.034		
						Triglycerides	↑ 0.22 *P* < 0.00		
Pillinger et al. [54]	Cardiometabolic profile	20	2003–2016	Minimal (<2 weeks)	1167/1184	Total cholesterol	↓ −0.19 *P* = 0.005	29–77%	10/11
						HDL cholesterol	↔ −0.22 *P* = 0.065		
						LDL cholesterol	↓ −0.22 *P* = 0.001		
						Triglycerides	↑ 0.14 *P* < 0.05		
						Leptin	↔ 0.05 *P* = 0.779		
Flatow et al. [15]	Cardiometabolic profile	18	1996–2012	Not naive	535/615	Plasma TAS	↓ −1.42 *P* < 0.01	No *I*^2^ values. χ^2^ *P* value range <0.01–0.91	7/11
						Serum TAS	↓ −1.12 *P* < 0.01		
						RBC CAT	↓ −0.48 *P* < 0.01		
						RBC GSH-Px	↔ 0.18 *P* = 0.26		
						RBC SOD	↓ −0.79 *P* < 0.01		
						Plasma SOD	↑ 0.45 *P* < 0.01		
						Plasma MDA	↑ 2.36 *P* < 0.01		
						Plasma TBARS	↑ 0.88 *P* < 0.01		
						Plasma nitrite	↓ −0.70 *P* < 0.01		
						Plasma uric acid	↓ −0.55 *P* < 0.01		
Upthegrove et al. [17]	Immune profile	23	1990–2012	Antipsychotic naive	570/683	IL-1β	↑ 1.17 *P* < 0.0001	DNA	6/11
						IL-2	↔ −0.20 *P* = 0.772		
						sIL-2R	↑ 1.34 *P* < 0.0001		
						IL-4	↑ 0.20 *P* = 0.861		
						IL-6	↑ 2.21 *P* = 0.013		
						TNF-α	↑ 0.94 *P* < 0.0001		
						IFN-γ	↔ 0.24 *P* = 0.760		
Goldsmith et al. [18]	Immune profile	24	1989-2014	Not naive	1393/1497	IL-1β	↑ 1.25 *P* < 0.01	0–97%	9/11
						IL-1RA	↑ 0.29 *P* < 0.01		
						IL-2	↔ 0.08 *P* = 0.48		
						sIL-2R	↑ 1.04 *P* < 0.01		
						IL-4	↓ −0.63 *P* < 0.01		
						IL-6	↑ 1.16 *P* < 0.01		
						IL-8	↑ 1.75 *P* < 0.01		
						IL-10	↑ 0.18 *P* = 0.01		
						IL-12	↑ 0.26 *P* = 0.02		
						IL-17	↔ 0.00 *P* = 0.99		
						IL-18	↔ 0.08 *P* = 0.28		
						TNF-α	↑ 0.31 *P* < 0.01		
						TGF-β	↑ 0.58 *P* < 0.01		
						IFN-γ	↑ 0.23 *P* < 0.01		
Miller et al. [19]	Immune profile	13	1989-2010	Antipsychotic naive/free (sub-group)	481/633	IL-1β	↑ 0.60 *P* < 0.001	60–98%	8/11
						IL-2	↔ −0.09 *P* = 0.44		
						sIL-2R	↑ 1.03 *P* < 0.001		
						IL-6	↑ 1.40 *P* < 0.001		
						IL-12	↑ 0.98 *P* < 0.001		
						TNF-α	↑ 0.81 *P* < 0.001		
						TGF-β	↑ 0.48 *P* < 0.001		
						IFN-γ	↑ 0.57 *P* = 0.001		
Fernandes et al. [20]	Immune profile	6	2007-2014	Antipsychotic naive/free	348/360	C-reactive protein	CRP: 0.63 *P* = 0.038	87%	9/11
Miller et al. [21]	Immune profile	5	1990-2008	Antipsychotic naive	125/323	Total lymphocytes	↑ 0.77 *P* < 0.01	0–57%	7/11
						CD3 lymphocytes	↑ 0.72 *P* < 0.01		
						CD4 lymphocytes	↑ 0.86 *P* < 0.01		
						CD8 lymphocytes	↔ 0.44 *P* = 0.10		
						B lymphocytes	↔ 0.30 *P* = 0.13		
Berger et al. [22]	HPA axis profile	6	2008–2015	Not naive	251/216	Cortisol awakening response	↓ −0.54 *P* < 0.001	24%	10/11
Chaumette et al. [23]	HPA axis profile	6	2007–2014	Not naive	215/226	Basal cortisol	↔ −0.15 *P* = 0.56	77%	10/11
Girshkin et al. [24]	HPA axis profile	10	1996–2013	Not naive	285/282	Basal cortisol	↔ −0.10 *P* = 0.644	83%	9/11
Gonzalez-Blanco et al. [25]	HPA axis profile	8	1990–2014	Minimal (<1 week)	Male 141/191	Prolactin	↑ 1.02 *P* < 0.001	81%	10/11
		6			Female 67/116	Prolactin	↑ 0.43 *P* < 0.01	66%	9/11

*HC* healthy control, *OGTT* oral glucose tolerance test, *FG* fasting glucose, *IR* insulin resistance, *TC* total cholesterol, *LDL* low-density lipoprotein, *HDL* high-density lipoprotein, *TG* triglyceride, *TAS* total antioxidant status, *RBC* red blood cell, *CAT* catalase, *GSH-Px* glutathione peroxidase, *SOD* superoxide dismutase, *MDA* malondialdehyde, *TBARS* thiobarbituric acid reactive substances, *IL* interleukin, *TNF-α* tumour necrosis factor-α, *IFN- γ* interferon-γ, *TGF- β* transforming growth factor-β, *CAR* cortisol awakening response, *AMSTAR* a measurement tool to assess systematic reviews

**Table 2:** Meta-analyses examining CNS alterations in first-episode psychosis that met inclusion criteria

Meta-analysis	Objective	No. of studies	Study range	Anti-psychotic status	Patient/HC no.	CNS parameter	Effect size	Hetero-geneity (*I*^2^/Q)	AMSTAR
Bora et al. [56]	Structural	13	2003–2010	Not naive	415/459	Superior temporal gyrus	↓ −0.29 *P* < 0.000005	Not specified	7/11
					415/459	Right dorsal anterior cingulate	↓ −0.24 *P* = 0.0002		
		4	2006–2010		127/120	Fractional anisotropy reduction: L temporal WM	↓ −0.40 *P* = 0.00004		
					127/120	Fractional anisotropy reduction: R PLIC	↓ −0.34 *P* = 0.0003		
Adriano et al. [40]	Structural	13	1998–2010	Not naive	388/562	Right Hippocampal volume	↓ −0.56 *P* < 0.00001	16%	7/11
					388/562	Left Hippocampal volume	↓ −0.60 *P* < 0.00001	56%	
Adriano et al. [42]	Structural	15	1999–2009	Not naive	173/211	Right Thalamus volume	↓ −0.45 *P* < 0.0001	0%	6/11
					173/211	Left Thalamus Volume	↓ −0.48 *P* < 0.0001	0%	
Haijma et al. [43]	Structural	15	1998–2011	Antipsychotic naive	364/490	Total brain volume	↓ −0.21 *P* = 0.003	0%	5/11
		10			238/292	Total gray matter	↓ −0.36 *P* = 0.000066	0%	
		7			182/286	Total CSF	0.31 *P* = 0.011	30%	
		8			194/251	Hippocampal volume	↓ −0.43 *P* = 0.0000076	0%	
		7			152/260	Thalamus volume	↓ −0.68 *P* = 0.00083	67%	
		10			299/422	Caudate nucleus	↓ −0.38 *P* = 0.00000095	0%	
Vita and de Peri [44]	Structural	7	1990–2006	Not naive	290/355	Right hippocampal volume	↓ −0.36 *P* < 0.05	Not specified	4/11
						Left hippocampal volume	↓ −0.57 *P* < 0.05		
de Peri et al. [45]	Structural	21	1991–2011	Not naive	686/772	Total brain volume	↓ −0.26 *P* < 0.001	*Q* = 34.21 *P* = 0.02	6/11
		12			412/438	Total gray matter	↓ −0.36 *P* < 0.001	*Q* = 13.23 *P* = 0.27	
		8			308/319	Lateral ventricles (total)	↑ 0.38 *P* < 0.001	*Q* = 3.62 *P* = 0.82	
		12			396/429	Right lateral ventricle	↑ 0.40 *P* < 0.001	*Q* = 7.57 *P* = 0.75	
		12			396/429	Left lateral ventricle	↑ 0.49 *P* < 0.001	*Q* = 11.09 *P* = 0.37	
Vita et al. [46]	Structural	11	1991–2003	Not naive	340/422	Total brain volume	↓ −0.24 *P* = 0.002	Not specified	5/11
		8			241/206	Right lateral ventricle	↑ 0.47 *P* < 0.0001		
		8			241/206	Left lateral ventricle	↑ 0.61 *P* < 0.0001		
		4			114/102	Total lateral ventricle	↑ 0.32 *P* = 0.022		
		6			204/162	Third ventricle	↑ 0.59 *P* < 0.0001		
		4			121/101	Right temporal lobe	↔ −0.07 *P* = 0.617		
		4			121/101	Left temporal lobe	↔ −0.15 *P* = 0.258		
		6			187/268	Right hippocampus	↓ −0.47 P < 0.0001		
		6			187/268	Left hippocampus	↓ −0.66 P < 0.0001		
Fusar-Poli et al. [47]	Structural	8	Not specified	Antipsychotic naive	206/202	Total gray matter	↓ −0.83 P < 0.001	9%	5/11
					206/202	Superior temporal gyrus gray matter volume	↓ −0.56 P < 0.00005		
Erickson et al. [48]	Neuro-physiologic	13	1999–2015	Not naive	331/393	Mismatch negativity amplitude	↑ 0.42 *P* < 0.05	Not specified	5/11
Qiu et al. [49]	Neuro-physiologic	17	1998–2009	Not naive	569/747	P300 amplitude	↓ −0.83 *P* = 0.00001	55%	8/11
		16			506/747	P300 latency	↑ 0.48 *P* = 0.005	86%	
Chen et al. [50]	Neuro-functional	4	2003–2014	Antipsychotic naive	105/214	P300 latency	−0.13 *P* = 0.31	‘Not significant’	4/11
						P300 amplitude	↔ 0.48 *P* = 0.05		
Haigh et al. [51]	Neuro-physiologic	9	2002–2013	Not naive	242/395	Pitch deviant MMN	↔ −0.04 *P* > 0.05	Not specified	4/11
		10			360/531	Duration deviant MMN	↓ −0.47 *P* < 0.05		
Brugger et al. [52]	Neuro-chemical	19	1997–2009	Not naive	376/428	Frontal NAA levels	↓ −0.45 *P* < 0.0001	49%	5/11
		11			232/189	Temporal NAA levels	↓ −0.53 *P* = 0.0025	63%	
		5			102/88	Thalamus NAA levels	↓ −0.40 *P* = 0.0203	23%	
		6			125/91	Basal Ganglia NAA levels	↔ −0.09 *P* = 0.599	24%	

*NAA N*-acetyl aspartate, *MMN* mismatch negativity, *WM* white matter, *PLIC* posterior limb of the internal capsule, *CSF* cerebrospinal fluid, *L* left, *R* right, *AMSTAR* a measurement tool to assess systematic reviews

As well as individual meta-analyses being run for each parameter as described, six separate subgroup meta-analyses were performed examining data for overall immune, cardiometabolic, HPA, brain structural, neurophysiological, and neurochemical systems. Subgroup summary effect size magnitudes were calculated by running a combined analysis of all studies assigned to a subgroup (e.g., to calculate the summary effect size magnitude for immune alterations, a single analysis was performed that combined IL-1β, sIL-2R, IL-6, TNF-α, TGF-β, CRP, and total lymphocyte count data sets). If a single study provided results for more than one subgroup parameter (e.g., data for several cytokines from a single study population), then the patient and control numbers for that study were divided by the number of parameters contributed to the summary meta-analysis (e.g., if two different cytokines were reported by a single study, then the population number for that study was divided by 2). Using the same methodology, overall meta-analyses for CNS and non-CNS alterations were calculated. Sensitivity analyses for antipsychotic naive FEP were performed.

### Statistical comparison of effect sizes for central nervous system and non-central nervous system alterations in first-episode psychosis

After obtaining effect size estimates for each CNS (brain structural, neurophysiological, and neurochemical) and non-CNS system (immune, cardiometabolic, and HPA), we next performed bivariate comparisons of each of these six effect sizes against one another using a Wald-type test. We determined statistical significance by entering each pair of effect size estimates into a fixed effects model (given that the residual heterogeneity had previously been accounted for in the initial random effects meta-analyses). *P* < 0.05 was deemed significant. This method was also used to compare overall summary CNS and non-CNS effect sizes. Sensitivity analyses were performed restricting analyses to antipsychotic naive cohorts. All statistical tests were conducted using the metafor package [39] in the R statistical programming language.

## Results

For non-CNS dysfunction, of 365 citations retrieved, 15 meta-analyses met inclusion criteria [11–25] (Table 1). For CNS dysfunction, of 446 citations retrieved, 13 meta-analyses met inclusion criteria [40–52] (Table 2). Data were extracted from a total of 165 case–control studies (eAppendix 3). After excluding overlapping samples, the total sample size was 13,440 (6806 patients and 6634 controls), with 6817 in the non-CNS sample (3300 patients and 3517 controls), and 6623 in the CNS sample (3506 patients and 3117 controls). The quality of studies was medium to high (AMSTAR scores 6–10, Tables 1 and 2).

### Meta-analytic outcomes for central nervous system and non-central nervous system alterations in first-episode psychosis

**Fig. 1:**
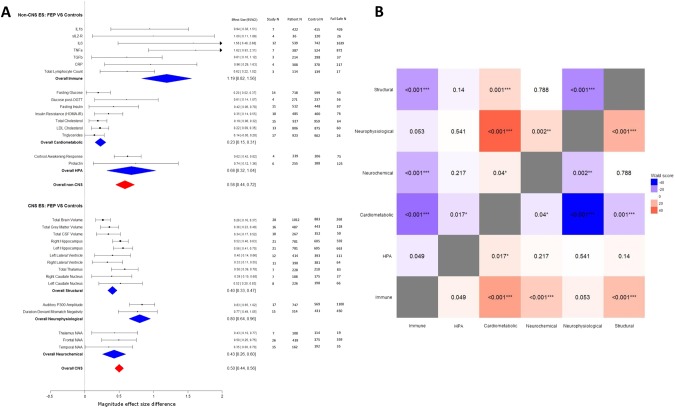
An overview and comparison of CNS and non-CNS alterations in first-episode psychosis. Figure 1a: Forest plot for magnitude of immune, cardiometabolic, HPA, brain structural, neurophysiological, and neurochemical alterations in first-episode psychosis compared with healthy controls. Each line represents a summary effect size for a meta-analysis in one parameter: squares represent the summary effect size for that parameter, with the horizontal line running through each square illustrating the width of the overall 95% CI. Blue diamonds represent summary effect sizes for immune, cardiometabolic, HPA, structural, neurophysiological, and neurochemical systems: the middle of each diamond represents the summary effect size, and the width of the diamond depicts the width of the overall 95% CI. Red diamonds represent summary effect sizes and accompanying 95% CI for non-CNS and CNS effect sizes. ES effect size, CNS central nervous system, FEP first-episode psychosis, HPA hypothalamic pituitary adrenal axis, IL1β interleukin-1β, sIL2-R soluble interleukin-2 receptor, IL6 interleukin-6, TGFβ transforming growth factor-β, CRP C-reactive protein, NAA *N*-acetylaspartic acid, N number. Figure 1b: Heat map comparing relative magnitude of effect sizes (ES) for immune, hypothalamic pituitary adrenal (HPA) axis, cardiometabolic, brain structural, neurophysiological, and neurochemical alterations in first-episode psychosis (FEP). The map is read from left to right, comparing parameters on the *y* axis with parameters on the *x* axis. A negative Wald score (blue squares) demonstrates that the parameter ES on the *y* axis is numerically lower compared with the intersecting parameter ES on the *x* axis. A positive Wald score (red squares) demonstrates that the parameter ES on the *y* axis is numerically higher than the intersecting parameter ES on the *x* axis. Numbers within the squares are the *P* values that accompany the Wald score, e.g., structural abnormalities show significantly smaller patient-control differences compared to immune abnormalities, and significantly greater differences compared to cardiometabolic abnormalities

Figure 1a depicts a forest plot for magnitude of immune, cardiometabolic, HPA, brain structural, neurophysiological, and neurochemical alterations in first-episode psychosis compared with healthy controls, as well as overall summary effect sizes for CNS and non-CNS alterations. As per Cohen’s guidelines [53], a medium overall summary effect size for non-CNS alterations in FEP was observed, (*g = *0.58 (95% CI: 0.44–0.72). A medium overall summary effect size for CNS alterations in FEP was also observed (*g = *0.50 (95% CI: 0.44–0.56). Similar results were observed in antipsychotic naive sensitivity analyses (effect size for non-CNS alterations: *g = *0.51 (95% CI: 0.34–0.67); effect size for CNS alterations: *g = *0.48 (95% CI: 0.39–0.58), eFigure 3).

### The immune system

Five meta-analyses examining immune disturbances in FEP were identified [17–21]. Four meta-analyses examined inflammatory mediators [17–20], and one lymphocyte counts [21]. After allowing for overlapping studies, data were extracted for a total sample size of 1343 patients and 1643 controls. FEP is associated with elevated blood cytokine levels, specifically IL-1β, sIL2R, IL-6, TNFα, TGFβ, CRP, and elevated total lymphocyte counts (effect size range: 0.61–1.62) (Fig. 1a). The summary effect size of immune alterations in FEP is 1.19 (95% CI: 0.82–1.56). Fail-safe N calculations demonstrated that between 17 and 1639 additional negative studies would be required for these findings to lose significance. Heterogeneity of studies was low to high (*I*^2^: 0–98%), and study quality medium to high (AMSTAR: 6–9). Antipsychotic naive FEP is associated with elevated blood cytokine levels, specifically IL-1β, sIL2R, IL-6, and TNFα (effect size: 1.00–1.86). The overall effect size for magnitude of immune alterations in antipsychotic naive FEP is 1.46 (95% CI: 0.74–2.18) (eFigure 3).

### The cardiometabolic system

Six meta-analyses examining cardiometabolic dysfunction in FEP were identified [11–16]. Three meta-analyses focused on glucose and insulin disturbance [11–13], two on lipid disturbance [14, 16], and one on oxidative stress [15]. After allowing for overlapping studies, data were extracted for a total sample size of 1556 patients and 1480 controls. All data extracted were for antipsychotic naive individuals. Antipsychotic naive FEP is associated with elevated fasting glucose, glucose following the oral glucose tolerance test, fasting insulin, and insulin resistance (effect size range 0.20–0.61), raised triglycerides (effect size: 0.14), and reduced total cholesterol and LDL cholesterol (effect size range: −0.22 to −0.19) (Fig. 1a). We were unable to extract sufficient data to allow for a meta-analysis of oxidative stress parameters in FEP. The summary effect size of cardiometabolic alterations in antipsychotic naive FEP is 0.23 (95% CI: 0.15–0.31). Fail-safe N calculations demonstrate that between 26 and 97 additional negative studies would be required for findings to lose significance. Heterogeneity was low to high (*I*^2^: 0–97%), and study quality medium to high (AMSTAR: 7–10).

### The hypothalamic–pituitary–adrenal system

Four meta-analyses examining HPA dysregulation in FEP were identified [22–25]. Two meta-analyses reported on morning cortisol [23, 24], one on cortisol awakening response [22], and one on prolactin levels [25]. Prolactin levels were included as a marker of HPA axis activation owing to previous evidence that its levels increase in response to various stressors, with a direct correlation observed between prolactin levels and both adrenocorticotrophic hormone and cortisol levels in healthy controls [54]. Data were extracted for a total sample size of 401 patients and 394 controls. The cortisol awakening response is blunted in FEP (effect size: 0.62), and prolactin levels are elevated in antipsychotic naive FEP [25] (effect size: 0.74) (Fig. 1a). The summary effect size of HPA alterations in FEP is 0.68 (95% CI: 0.32–1.04). Fail-safe N calculations demonstrate that between 75 and 125 negative studies would be required for these findings to lose significance. Heterogeneity was low to high (*I*^2^: 24–83%), and study quality high (AMSTAR: 9–10). There were insufficient data to allow for a meta-analysis of cortisol awakening response in antipsychotic naive FEP.

### Central nervous system: brain structural changes

Eight meta-analyses examining brain structure in FEP were identified [40, 42–47, 55]. Data were extracted for a total sample size of 1937 patients and 1656 controls. FEP is associated with reductions in both total and regional brain volumes (effect size: 0.26–0.58), and an increase in CSF volume (effect size: 0.34) (Fig. 1a). The summary effect size of brain structural alteration in FEP is 0.40 (95% CI: 0.33–0.47). Fail-safe N calculations demonstrate that between 27 and 663 additional negative studies would be required for these findings to lose significance. Heterogeneity was low to medium (*I*^2^: 9–56%), and study quality medium (AMSTAR: 4–7). Antipsychotic naive FEP is also associated with total and regional brain volume reduction (effect size: 0.23–0.87), and an in increase in total CSF volume (effect size: 0.32). The summary effect size of brain structural alterations in antipsychotic naive FEP is 0.44 (95% CI: 0.34–0.54) (eFigure 3).

### Central nervous system: neurophysiological changes

Four meta-analyses examining neurophysiological changes in FEP were identified [48–51]. Data were extracted for a total sample size of 1051 patients and 980 controls. FEP is associated with decreased auditory P300 amplitude (effect size: 0.83), and reduced duration-deviant mismatch negativity (effect size: 0.77) (Fig. 1a). The summary effect size for magnitude of neurophysiological alteration in FEP is 0.80 (95% CI: 0.64–0.96). Fail-safe N calculations demonstrate that between 450 and 1100 additional negative studies would be required for these findings to lose significance. Sample heterogeneity was medium to high (*I*^2^: 55–86%), and study quality medium to high (AMSTAR: 4–8). Antipsychotic naive FEP is associated with a reduction in auditory P300 amplitude (effect size: 0.86) and latency (effect size: 0.63), as well as a reduction in duration-deviant mismatch negativity (effect size: 0.67). The overall effect size for magnitude of neurophysiological alterations in antipsychotic naive FEP is 0.70 (95% CI: 0.47–0.94) (eFigure 3).

### Central nervous system: neurochemical changes

One meta-analysis examining brain chemistry in FEP was identified [52]. Data were extracted for a total sample size of 518 patients and 481 controls. FEP is associated with decreased levels of *N*-acetyl aspartate concentrations across multiple brain regions (effect size: 0.35–0.50) (Fig. 1a). The overall effect size for magnitude of neurochemical alteration in FEP is 0.43 (95% CI: 0.26–0.60). Fail-safe N calculations demonstrate that between 19 and 359 additional negative studies would be required for these findings to lose significance. Sample heterogeneity was low to medium (*I*^2^: 23–63%), and study quality medium (AMSTAR: 5). Antipsychotic naive FEP is associated with a reduction in frontal cortical NAA (ES: 0.34) (eFigure 3).

### Statistical comparison of effect sizes for central nervous system and non-central nervous system alterations in first-episode psychosis

Overall, CNS and non-CNS effect sizes were compared using a Wald-type test. There was no significant difference between the overall effect size of alterations in the CNS compared with the overall effect size of alterations in the non-CNS systems examined (*P* = 0.283), and this remained the case when analyses were restricted to studies of antipsychotic naive patients (*P* = 0.825).

### Individual system comparisons

A heat map of respective Wald scores and associated significance values between each of the systems examined (immune, cardiometabolic, HPA, brain structural, neurochemical, and neurophysiological) was constructed to allow graphical representation of the relative effect size magnitude difference between systems (Fig. 1b). Wald test comparisons of overall immune effect size with CNS effect sizes in FEP demonstrated significantly higher effect size magnitudes for immune alterations compared with brain structural (*P* < 0.001) and neurochemical (*P* < 0.001) alterations, and no significant difference when compared with neurophysiological alterations (*P* = 0.053). For antipsychotic naive FEP, summary effect size for immune alterations was significantly higher in antipsychotic naive FEP compared with brain structural (*P* = 0.006) and neurochemical alterations (*P* = 0.005), and no significant difference when compared with neurophysiological alterations (*P* = 0.05) (eFigure 4). Comparisons of the summary effect size for cardiometabolic alterations with those for CNS alterations in antipsychotic naive FEP demonstrated significantly reduced effect size magnitudes for cardiometabolic alterations compared with brain structural (*P* = 0.001), neurochemical (*P* = 0.04), and neurophysiological alterations (*P* < 0.001). Comparisons of summary effect size of HPA with those for CNS alterations in FEP demonstrated no significant difference when compared with brain structural (*P* = 0.14), neurochemical (*P* = 0.217), and neurophysiological (*P* = 0.541) alterations.

### Correlations between non-central nervous system parameters and central nervous system parameters

In view of evidence of both CNS and non-CNS alterations in FEP, we examined evidence for associations between these parameters. Only a limited number of observational studies have investigated relationships between CNS and non-CNS measures in FEP. Studies in broader psychotic illness, including schizophrenia, present data that is conflicting, which may reflect the heterogeneity of the population studied, but also the multi-faceted roles of the metabolic, immune, and endocrine parameters we have examined. For example, IL-6 has both neurodegenerative [56] and neuroprotective properties [57]. Thus, conflicting outcomes with regard to correlations between pro-inflammatory cytokines and brain structural alterations might be expected. Indeed, in schizophrenia, hippocampal volumes correlate directly with the pro-inflammatory cytokine interleukin-18 [58], while pro-inflammatory IL-1β titres correlate indirectly with Broca’s area volume in a “pro-inflammatory” subgroup of patients [59]. Thus, we have evidence of elevated pro-inflammatory cytokines being differentially associated with regional brain volume alterations: further studies are required to clarify regional brain structural alterations in the context of systemic inflammation in FEP. In terms of HPA axis alterations and influence on regional brain structure, although one study has failed to demonstrate a relationship between cortisol levels and hippocampal volume in FEP [60], Mondelli and colleagues have reported an inverse correlation between blood cortisol levels and hippocampal volumes [61], and the degree of left-sided hippocampal volume reduction has been associated with a blunted cortisol awakening response in FEP [62]. Beyond brain structural alterations, in schizophrenia, diffusion tensor imaging has demonstrated that peripheral IL-6 levels inversely correlate with measures of fractional anisotropy in the forceps major, inferior longitudinal fasciculus (ILF), and inferior fronto-occipital fasciculus (IFOF), a relationship not demonstrated in healthy controls. In both the ILF and IFOF, IL-6 levels correlate directly with radial diffusivity measures. CRP levels in schizophrenia also show an inverse correlation with fractional anisotropy within the forceps major [63]. Thus, in psychosis, selected neural pathways may be differentially susceptible to systemic immune alterations, although replication of these findings is required. We were unable to find any studies in FEP exploring correlations between CNS parameters and cardiometabolic disturbances. In individuals with type 2 diabetes who do not have a psychotic disorder, insulin resistance is associated with hippocampal atrophy and memory impairment [64]. Since cognitive impairment is a key feature of psychotic illness, and we have demonstrated that antipsychotic naive FEP is associated with both insulin resistance and reduced hippocampal volumes, future structural imaging studies combined with metabolic assays are required to clarify if a correlation between impaired glucose homeostasis and hippocampal structural alterations also exists in FEP.

### Correlations between non-CNS parameters and symptom severity

Although the evidence presented indicates that cardiometabolic, immune, and HPA alterations are present in early psychosis, this does not indicate whether these abnormalities are linked to the clinical expression of the disorder. As such, we also examined evidence of a “biological gradient” between these markers and symptom measures. In antipsychotic naive FEP, positive symptom severity, as assessed using the positive and negative syndrome scale (PANSS), correlates indirectly with fasting glucose levels [65] and insulin resistance [66], indicating more severe symptoms are associated with less marked glucoregulatory disturbance. As part of a meta-analysis examining CRP titres in schizophrenia performed by Fernandes and colleagues [67], meta-regression of effect size for CRP changes on PANSS-positive scores demonstrated that the greater the severity of positive symptoms, the greater the increase in CRP (*r* = 0.12; 95% CI: 0.03–0.23; *P* = 0.013). Broader evidence for a biological gradient between symptom severity and inflammatory cytokines in psychosis is however inconsistent and contradictory. For example, in antipsychotic naive FEP, levels of the anti-inflammatory cytokine IL-10 inversely correlate with PANSS-negative scores [68], indicating this anti-inflammatory marker is associated with less severe symptoms, however PANSS-positive scores in FEP have been observed to inversely correlate with pro-inflammatory IL-6 [69]. Studies in broader psychotic illness have demonstrated that IL-6 levels correlate directly with total psychopathology [70, 71], however others have failed to demonstrate an association [72–74]. No significant correlations have been reported between TNF-α, IL-1β, IL-12, and TGF-β levels with symptomatology in FEP [75–78]. Correlations between HPA axis alterations and symptom severity in FEP are similarly inconsistent and contradictory. For example, although cortisol levels have been observed to directly correlate with symptom severity [79–81], there have been negative studies [82–84], and inverse correlations with illness severity reported [85]. Varied outcomes may be a consequence of heterogeneity of patient populations and parameter measurement techniques between studies. Future projects may benefit from recruitment of a more homogenous group of participants, either by applying more stringent diagnostic inclusion criteria (e.g., focussing on individuals with predominant negative or positive symptoms, as demonstrated by Kirkpatrick and colleagues who defined differences in glucose tolerance between deficit and non-deficit schizophrenia) [86] or by stratifying patients based on a pre-defined physiological parameter (e.g., a “pro-inflammatory” group based on cytokine titres) [59].

## Discussion

**Fig. 2:**
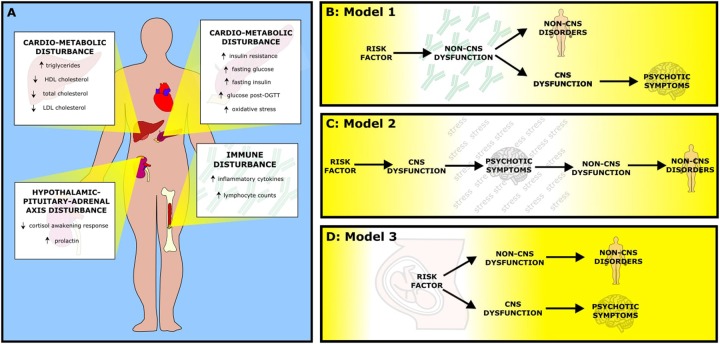
A summary of non-CNS alterations in first-episode psychosis, and a consideration of potential pathoetiology. Figure 2a: First-episode psychosis shows alterations in multiple systems in addition to the central nervous system. OGTT oral glucose tolerance test, HDL high-density lipoprotein, LDL low-density lipoprotein. Figure 2b–d: Models of the relationship between psychosis and non-CNS dysfunction. Figure 2b: Model 1: A risk factor induces non-CNS dysfunction, which may consequently impact CNS function to increase the risk of psychosis. Figure 2c: Model 2: A risk factor induces CNS dysfunction and thence psychotic symptoms, which may consequently trigger non-CNS dysfunction. Figure 2d: Model 3: A shared risk factor may result in the development of psychosis and non-CNS dysfunction through independent mechanisms. CNS central nervous system

The evidence analyzed in this review, compiled from 165 case–control studies and an overall sample size of 13,440 subjects, indicates that there are a range of significant non-CNS as well as CNS alterations in patients with first-episode psychosis (summarized in Fig. 2a), and that the overall magnitudes of alteration for CNS and non-CNS alterations are not significantly different. Fail-safe N calculations, which provide a surrogate marker of how robust these findings are, demonstrate that large numbers of null studies would need to be added to these meta-analyses for both CNS and non-CNS outcomes to lose statistical significance (fail-safe N ranges: immune 17–1639; HPA 75–125; cardiometabolic 26–97; brain structural 27–663; neurophysiological 450–1100; neurochemical 19–359). Although our review of observational studies in psychosis suggests that there may be a link between certain non-CNS and CNS alterations as well as some non-CNS parameters and symptom severity, the number of these correlative studies is small and the results are inconsistent, making definitive inferences difficult.

### Strengths and limitations

The major strength of this meta-review is its focus on FEP and antipsychotic naive data. Thus, where possible the confounding effects of illness chronicity and treatment were limited. There are of course limitations to studies examining physical dysfunction in FEP (see limitations box). It is important to recognize that the FEP population is inevitably diagnostically heterogenous. To investigate the degree of heterogeneity, we extracted the diagnoses of patients from each study where reported (see eAppendix4). This shows that the overall proportion of FEP patients included in our analyses who had a diagnosis of schizophrenia was 74% in the non-CNS group and 84% in the CNS group. While some of the patients without a diagnosis of schizophrenia will go on to develop schizophrenia, it still important to recognize that some will never develop schizophrenia and, consequently, our findings should not be taken as being specific to schizophrenia. While the difference in the proportion with schizophrenia between groups is modest, we cannot exclude that this has moderated the effect sizes. Thus, we recommend future FEP studies include follow-up to determine diagnoses, and report findings separately by diagnosis to enable this to be tested. The specificity of findings is also low, with other serious mental illnesses showing similar physiological dysfunction [87, 88]. However, it should be recognized that many of the same factors apply to CNS alterations, and reverse causality is possible, with lifestyle factors potentially impacting both CNS and non-CNS measures. For example, smoking [89], stress [90], drug [91], and alcohol [92] abuse are associated with gray matter volume loss. Despite our focus on early psychosis, studies often fail to report or examine the impact of duration of untreated psychosis. As such, it remains possible that non-CNS changes are secondary to emerging psychotic symptoms, e.g., due to resultant poor lifestyle habits, social isolation, and stress [93, 94]. Moreover, inconsistencies in studies investigating a “biological gradient” between CNS and non-CNS alterations, and non-CNS alterations with symptom severity, limit arguments regarding non-CNS alterations contributing to pathological causality in psychosis as per Bradford Hill’s guidelines [95]. Although meta-analysis as a methodology is a powerful tool to summarize research knowledge in a field, it has limitations [96]. These limitations relate to the reliability of a meta-analysis’ outcome being dependent on the quality of the data meta-analyzed, the size of the samples included, the potential for type 1 error owing to publication bias toward “positive” results, and the heterogeneity of study populations included. However, our quality assessment of meta-analyses selected for this review was reassuring, with only medium-high quality analyses included. Although heterogeneity of studies documented by these meta-analyses varied, our meta-analyses used a random-effects model which is robust to heterogeneity. The fail-safe N approach has limitations [97]. For example, the formula assumes that the mean effect of unpublished null studies is zero, whereas studies may also have negative effect sizes, thereby requiring fewer studies to nullify the mean effect. However, the fail-safe N was applied across organ systems, thus, assuming there is similar publication bias across CNS and non-CNS research fields in psychosis, we can be confident that the fail-safe N is a reasonable quantitative measure by which the relative strength of the evidence supporting alterations in CNS and non-CNS parameters can be assessed. Indeed, fail-safe N calculations suggest outcomes for certain CNS and non-CNS parameters are more robust than others (Fig. 1a). Of the 15 comparative Wald analyses between CNS and non-CNS alterations in FEP, only four contrasts changed significance (based on *α* < 0.05) when moving from medicated to antipsychotic naive sensitivity analyses. There was no difference overall between CNS and non-CNS effect size magnitudes in both medicated and antipsychotic naive cohorts. This suggests that antipsychotic treatment does not significantly moderate effect sizes documented, providing further evidence that CNS and non-CNS alterations co-occur in FEP.

### The putative nature of the relationship between central nervous system and non-central nervous system abnormalities in first-episode psychosis

Figure 2b–d illustrates three putative models that could explain our findings of both non-CNS and CNS abnormalities in FEP. Model 1 (Fig. 2b) shows how a risk factor inducing non-CNS dysfunction may lead to development of non-CNS disorders as well as impacting CNS function leading to psychosis. An example is that of paraneoplastic-induced anti-*N*-methyl-D-aspartate (NMDA)-receptor encephalitis, which is associated with psychosis. Resection of the causative tumor is associated with resolution of psychotic experiences [98]. More broadly, immune dysregulation could be responsible for co-development of psychosis and cardiometabolic disease. Pre-clinical studies demonstrate that peripheral inflammation can induce neuro-inflammation [99], which could potentially contribute to the pathogenesis of psychosis [100]. There is also evidence of inflammatory cytokines modulating dopaminergic and glutamatergic neurotransmission [101, 102]. As dysfunction in these neurotransmitters is implicated in the development of psychosis [103], this could link cytokine alterations to development of psychosis. As chronic inflammation is associated with accelerated atherosclerotic plaque formation, insulin resistance, and increased cardiovascular risk [36], elevated cytokines in psychosis could also be playing a role in the increased risk of cardiovascular disease in this population. Altered lipid turnover both peripherally and centrally may also be a consequence of an inflammatory process in FEP. Total and LDL cholesterol are reduced in antipsychotic naive FEP compared with healthy controls, a finding that is maintained in body mass index (BMI) sensitivity analyses [14, 16]. It has been hypothesized that the pro-inflammatory state of FEP is responsible for a “paradoxical” reduction in cholesterol via a similar mechanism seen in inflammatory arthritides [16]. Whether a similar mechanism modulates CNS lipid metabolism in schizophrenia and thus plays a role in disease pathogenesis through resultant synaptic dysfunction remains unclear. Such a mechanism would fit with model 1. In contrast, model 2 (Fig. 2c) shows how a risk factor can induce CNS dysfunction resulting in psychotic symptoms, which then trigger non-CNS dysfunction. An example is that the stress of psychosis could lead to HPA axis activation. Supporting this, psychological stress increases cortisol levels [104]. Cortisol excess is associated with hypertension, obesity, insulin resistance, dyslipidemia, and cardiovascular disease [105]. Thus, hypercortisolemia associated with psychosis may be contributing to cardiometabolic disease. It should however be recognized that, in addition to causing non-CNS dysfunction, stress may also contribute to the development of psychosis [106], suggesting a model whereby an exposure contributes jointly to CNS and non-CNS dysfunction. Finally, model 3 (Fig. 2d) proposes that a common risk factor has independent and parallel effects that result in the separate development of psychosis and non-CNS dysfunction. For example, population-based cohort studies have demonstrated that cardiometabolic disease [107] and psychosis share risk factors, including low-birth weight [108], pre-term birth [109, 110], and maternal malnutrition [111, 112]. Nutritional deficiencies in utero may result in neurodevelopmental changes increasing vulnerability to psychosis [113]. Nutritional deficiencies may also result in epigenetic changes relating to metabolic function, leading to metabolic alterations and ultimately diabetes [114]. Similarly, there may be shared genetic risk between psychosis and non-CNS disturbances. Genome-wide association studies have demonstrated pleiotropic enrichment between genes conferring risk for schizophrenia and non-CNS alterations, including immune and metabolic processes [115, 116].

### Critique and comparison of the models

The models discussed above are intended to summarize the main potential relationships that exist between CNS and non-CNS alterations in psychosis, and it should be recognized that there is evidence for and against each model. The relative strengths and weakness of the three models proposed are summarized in Supplementary Information (eBox 1). Model 1 is supported by cases where a non-CNS disorder clearly predates psychosis, which resolves when the non-CNS disorder is treated, such as the example of NMDA auto-antibodies leading to psychosis discussed above, but these cases are rare [98]. Moreover, population-based cohort studies have observed that elevated levels of serum CRP [117] and IL-6 [118] in childhood are associated with increased risk of psychotic experience and schizophrenia in adulthood. Furthermore, the North American Prodrome Longitudinal Study (NAPLS) [119] has provided evidence that hypercortisolemia, inflammation, and elevated oxidative stress in individuals already deemed to be at risk for developing psychosis are risk factors for transition to FEP. However, many associations between non-CNS dysfunction and psychosis have not shown evidence of causation to date. To demonstrate causality, non-CNS dysfunction needs to be addressed prior to the onset of psychosis, for example in the prodrome, and show that the development of psychosis is prevented. In addition, although model 1 explains rare cases of psychosis, it is unlikely to account for typical cases of schizophrenia where CNS alterations are thought to occur early in neurodevelopment [106], unless non-CNS alterations also occur very early in development. Model 2, where non-CNS dysfunction emerges as a consequence of psychosis, is supported by meta-analytic evidence that resolution of acute psychosis is associated with normalization of previously elevated cytokines (IL-1β, IL-6, and TGF-β) [19]. Also, there is only limited evidence for alterations of certain non-CNS parameters in the prodrome (e.g., glucose and lipid disturbances), suggesting their later development may be a consequence of psychosis or its treatment. However, as described in support of model 1, several non-CNS alterations have been demonstrated in the prodrome. In addition, the observed reduction in levels of cytokines in association with the resolution of an acute psychotic episode could be part of the therapeutic action of treatment (supportive of model 1 rather than 2). Model 3, where a shared risk factor plays a role in development of psychosis and non-CNS alterations through divergent mechanisms, is supported by the lack of consistent relationships between a number of CNS and non-CNS alterations. Also, the heterogeneity in both non-CNS and CNS findings between patients could suggest divergent mechanisms underlie them [120]. However, we have identified certain correlations, e.g., between glucose dysregulation [65], insulin resistance [66], and PANSS-positive scores in FEP, that point toward these non-CNS alterations being linked with the clinical expression of psychosis, consistent with, although not proving, a common pathoetiological mechanism. Moreover, there is a paucity of studies testing relationships between non-CNS and CNS alterations in psychosis, limiting any conclusions regarding common causality. Overall, there is both supportive and contradictory evidence for all three models, and aspects of each model that remain to be fully tested. The contradictory evidence suggests that one model is unlikely to account for all cases of psychosis. Moreover, while the models provide an overarching framework, the specific mechanisms that might link risk factors, CNS and non-CNS alterations need to be investigated. Further work is clearly needed, particularly to investigate the causal nature of relationships, and how common they are to patients in general.

### Does the involvement of multiple systems in first-episode psychosis indicate that psychosis is a multisystem disorder?

Conceptually, multisystem disorders can be categorized into two groups: [121] (1) conditions where multiple organ systems are pervasively affected with no single predominant organ involved (e.g., inborn errors of metabolism); (2) conditions where one organ system is predominantly affected, but where other organs may concurrently be involved (e.g., rheumatoid arthritis). Since psychosis is by definition a description of psychological phenomena, it would be hard to argue that psychosis meets the first definition. However, consistent with the second definition, the evidence reviewed above suggests that early in psychosis dysfunction is present across multiple organ systems. Given the large effect sizes and number of negative studies required for many of these to become no longer significant, these findings appear robust, at least as robust as for many of the brain structural/functional alterations seen in psychosis. Moreover, we found that there is some, albeit limited, evidence that non-CNS measures are linked to symptoms and CNS changes in FEP. This could be taken as suggesting that psychosis, and by extension schizophrenia, should be considered a multisystem disorder. However, the International Classification of Disease-11 2010 Steering Group discussion paper on multisystem diseases [121], defined these as “diseases that regularly manifest without involvement of a common single system and with concomitant major involvement of several systems”, and, on this basis, a disorder such as rheumatoid arthritis would not be considered multisystem because, while it affects multiple systems, its predominant manifestation is musculoskeletal. By the same token, given that psychosis by definition is a disorder of thought and behavior, it is clear that CNS dysfunction bears the most direct relationship with the clinical expression of the disorder. This argues against psychosis being a multisystem disorder. However, this argument is inherently circular because psychotic disorders are diagnosed solely on the basis of mental symptoms. Here, while the evidence reviewed above goes some way to showing the magnitude of non-CNS effects is similar, in some cases larger, than CNS effects, what is currently lacking is robust evidence that the changes in non-CNS systems have commensurate clinical impacts, for example on functioning, prognosis, or mortality. Evidence is also needed on the nature of the relationship between CNS and non-CNS changes: if non-CNS changes are found to be due to a common pathoetiology or risk factor (model 3) or lead to CNS changes (model 1), this could support psychosis being a multisystem disorder, while finding that non-CNS alterations are secondary to mental symptoms (model 2) would not. A consequence of this would potentially be that diagnostic and prognostic assessment might incorporate assessment of non-CNS organ dysfunction in addition to assessment of thought and behavior. For example, if the pathophysiology of FEP includes a hypercortisolemic, pro-inflammatory state, then a biomarker “fingerprint” of antipsychotic naive FEP could conceivably include evidence of HPA axis dysregulation (e.g., blunted cortisol awakening response) with raised peripheral cytokines (e.g., IL-6) as well as relevant measures of CNS function.

### Implications and future directions

Further research is required to elucidate whether non-CNS dysfunction is a cause or a consequence of psychosis. Longitudinal studies of non-CNS parameters starting in people at clinical high risk for psychosis and continuing through development of psychosis is a potential approach, and has the advantage of including a control group exposed to the same risk factors (those individuals who do not develop psychosis), as well as the research advantage that a number will develop other mental disorders. This group could help address another key question, which is the degree to which non-CNS alterations are specific to psychosis or are a common feature of a number of mental disorders, potentially consistent with a model whereby the stress of mental illness leads to changes in other systems [122] (model 2). Other key areas that our review has highlighted as requiring further work are the degree to which alterations in non-CNS systems are linked to psychotic symptoms, and other clinical outcomes, and whether common pathoetiological mechanisms underlie both CNS and non-CNS alterations.

In terms of clinical practice, the majority of excess mortality seen in schizophrenia is due to non-CNS causes, predominantly cardiovascular disease [8], and life expectancy in schizophrenia has failed to improve relative to the general population over recent decades [1]. Our findings of cardiometabolic, inflammatory, and HPA axis alterations in FEP suggest that processes underlying excess mortality are present early in schizophrenia and other psychotic disorders. One implication for clinicians is to routinely consider these systems in the assessment of psychotic disorders. Studies are needed to determine if addressing non-CNS alterations early reduces development of physical co-morbidity, and ultimately reduces mortality in psychotic disorders (see summary box).

## Conclusions

Abnormalities in multiple organ systems in addition to the CNS are seen at onset of psychotic disorders with similar magnitudes to those seen in the CNS. While the causal relationship between non-CNS and CNS alterations remains to be determined, this evidence indicates that psychosis involves multiple systems from illness onset, although is not sufficient to define it as a multisystem disorder.

**Competing interests**

Professor Howes has received investigator-initiated research funding from and/or participated in advisory/speaker meetings organized by Astra-Zeneca, Autifony, BMS, Eli Lilly, Heptares, Janssen, Lundbeck, Lyden-Delta, Otsuka, Servier, Sunovion, Rand, and Roche. Neither Professor Howes nor his family have been employed by or have holdings/a financial stake in any biomedical company. Drs Pillinger, D’Ambrosio, and McCutcheon report no financial relationships with commercial interests.

Box 1 | **Limitations**

1. First-episode psychosis samples are heterogenous, including patients with a number of different psychotic disorders. This limits extrapolation of these findings to particular disorders, although the majority of patients included met criteria for schizophrenia.
2. Included studies often fail to report duration of untreated psychosis, thus impact of emerging psychosis on physical health is not quantified.
3. Other serious mental illnesses, e.g., depression, show similar physiological alterations, therefore these findings may not be specific to psychosis.
4. According to fail-safe N calculations, certain findings (e.g., immune and neurophysiological outcomes) are more robust than others. Outcomes of certain systems (e.g., HPA axis and CNS neurochemistry) are limited by small sample sizes.
5. Meta-analysis as a methodology has limitations when small sample sizes are used, if study populations are heterogenous, if studies are of poor quality, and when publication bias exists.
6. Inconsistencies in demonstrating a “biological gradient” between CNS and non-CNS alterations, and non-CNS alterations with symptom severity, limit arguments regarding the non-CNS associations observed contributing to pathological causality.
7. Although aimed to be representative, the CNS and non-CNS systems reviewed were not exhaustive, and other non-CNS organ systems implicated in psychotic illness, e.g., the gastrointestinal system were not discussed. Similarly, indices of CNS function, e.g., cognitive function, were not discussed.

Box 2 | **Summary of findings and outstanding questions**

1. Non-CNS abnormalities occur with similar effect sizes as CNS abnormalities in FEP.
2. Non-CNS abnormalities may be a cause or consequence of CNS dysfunction in psychosis or an epiphenomenon. Clarification of the causal relationship is required.
3. The predominance of mental symptoms in psychosis currently argues against re-conceptualizing psychotic disorder as a multisystem disorder, but this is partly a function of the mental basis of the diagnosis.
4. Studies are needed to determine if addressing non-CNS dysfunction from illness onset may contribute to reducing schizophrenia’s excess mortality rates.

## Electronic supplementary material

Supplementary Information
