# Correction to: Is psychosis a multisystem disorder? A meta-review of central nervous system, immune, cardiometabolic, and endocrine alterations in first-episode psychosis and perspective on potential models

**DOI:** 10.1038/s41380-018-0275-2

**Published:** 2018-10-18

**Authors:** Toby Pillinger, Enrico D’Ambrosio, Robert McCutcheon, Oliver D. Howes

**Affiliations:** 1 IoPPN, King’s College London, De Crespigny Park, London, SE5 8AF UK; 2 MRC London Institute of Medical Sciences (LMS), Du Cane Road, London, W12 0NN UK; 3 Institute of Clinical Sciences (ICS), Faculty of Medicine, Imperial College London, Du Cane Road, London, W12 0NN UK

**Keywords:** Diagnostic markers, Physiology, Schizophrenia

Correction to: Mol Psychiatry; 10.1038/s41380-018-0058-9; published online: 9 May 2018

This article was originally published under standard licence, but has now been made available under a [CC BY 4.0] license. The PDF and HTML versions of the paper have been modified accordingly.

