# Supplementary material for: Is psychosis a multisystem disorder? A meta-review of central nervous system, immune, cardiometabolic, and endocrine alterations in first-episode psychosis and perspective on potential models
Source: Mol Psychiatry. 2018 May 9;24(6):776–94. doi: 10.1038/s41380-018-0058-9 (PMC6124651; doi:10.1038/s41380-018-0058-9)
Supplement: Supplementary file 1 — Supplementary Information [file 41380_2018_58_MOESM1_ESM.docx]

**SUPPLEMENTARY INFORMATION**

**CONTENTS**

Pages 2-3 eTable 1: PRISMA Checklist

Pages 4-6 eAppendix 1: Methods

Page 7 eFigure 1: Search process for meta-analyses examining non-CNS dysfunction in FEP

Page 7 eFigure 2: Search process for meta-analyses examining CNS dysfunction in FEP

Page 8 eTables 2 and 3: AMSTAR aggregate scores (CNS and non-CNS)

Page 9 eTable 4: Fail Safe N scores for CNS and non-CNS parameters

Page 10 eFigure 3: Forest plot for CNS and non-CNS alterations in antipsychotic naïve FEP

Page 11 eFigure 4: Heat map comparing relative ES magnitudes between CNS and non-CNS systems in antipsychotic naïve FEP

Page 12 eAppendix 2: AMSTAR Checklist

Page 13-25 eAppendix 3: Studies included in meta- and Wald-analyses

Page 26-29 eAppendix 4: Proportion of patients with a DSM diagnosis of schizophrenia

Page 30 eBox 1: Summary of strengths and weaknesses for models linking CNS and non-CNS alterations in psychosis

Page 31-33 eAppendix 5: a priori protocol

Page 34-35 References

**eTable 1: PRISMA (2009) CHECKLIST**

| Section/Topic | # | Checklist Item | Reported on page # |
| --- | --- | --- | --- |
| TITLE | | | |
| Title | 1 | Identify the report as a systematic review, meta-analysis, or both. | 1 |
| ABSTRACT | | | |
| Structured summary | 2 | Provide a structured summary including, as applicable: background; objectives; data sources; study eligibility criteria, participants, and interventions; study appraisal and synthesis methods; results; limitations; conclusions and implications of key findings; systematic review registration number | 2 |
| INTRODUCTION | | | |
| Rationale | 3 | Describe the rationale for the review in the context of what is already known. | 4 |
| Objective | 4 | Provide an explicit statement of questions being addressed with reference to participants, interventions, comparisons, outcomes, and study design (PICOS). | 4 |
| METHODS | | | |
| Protocol and registration | 5 | Indicate if a review protocol exists, if and where it can be accessed (e.g., Web address), and, if available, provide registration information including registration number. | Supplementary |
| Eligibility criteria | 6 | Specify study characteristics (e.g., PICOS, length of follow-up) and report characteristics (e.g., years considered, language, publication status) used as criteria for eligibility, giving rationale. | Supplementary |
| Information sources | 7 | Describe all information sources (e.g., databases with dates of coverage, contact with study authors to identify additional studies) in the search and date last searched. | 4 |
| Search | 8 | Present full electronic search strategy for at least one database, including any limits used, such that it could be repeated. | Supplementary |
| Study selection | 9 | State the process for selecting studies (i.e., screening, eligibility, included in systematic review, and, if applicable, included in the meta-analysis). | Supplementary |
| Data collection process | 10 | Describe method of data extraction from reports (e.g., piloted forms, independently, in duplicate) and any processes for obtaining and confirming data from investigators. | Supplementary |
| Data items | 11 | List and define all variables for which data were sought (e.g., PICOS, funding sources) and any assumptions and simplifications made. | 5-6 |
| Risk of bias in individual studies | 12 | Describe methods used for assessing risk of bias of individual studies (including specification of whether this was done at the study or outcome level), and how this information is to be used in any data synthesis. | n/a |
| Summary measures | 13 | State the principal summary measures (e.g., risk ratio, difference in means). | 5-6 |
| Synthesis of results | 14 | Describe the methods of handling data and combining results of studies, if done, including measures of consistency (e.g., I^2^) for each meta-analysis. | 6-7 |
| Risk of bias across studies | 15 | Specify any assessment of risk of bias that may affect the cumulative evidence (e.g., publication bias, selective reporting within studies). | 6 |
| Additional analyses | 16 | Describe methods of additional analyses (e.g., sensitivity or subgroup analyses, meta-regression), if done, indicating which were pre-specified. | 6-7 |
| RESULTS | | | |
| Study selection | 17 | Give numbers of studies screened, assessed for eligibility, and included in the review, with reasons for exclusions at each stage, ideally with a flow diagram. | eFigures 1 and 2 |
| Study characteristics | 18 | For each study, present characteristics for which data were extracted (e.g., study size, PICOS, follow-up period) and provide the citations. | Tables 1 and 2 |
| Risk of bias within studies | 19 | Present data on risk of bias of each study and, if available, any outcome level assessment (see item 12). | Tables 1 and 2 |
| Results of individual studies | 20 | For all outcomes considered (benefits or harms), present, for each study: (a) simple summary data for each intervention group (b) effect estimates and confidence intervals, ideally with a forest plot. | Tables 1 and 2, Figure 1 |
| Synthesis of results | 21 | Present results of each meta-analysis done, including confidence intervals and measures of consistency. | Figure 1  and efigure 3 |
| Risk of bias across studies | 22 | Present results of any assessment of risk of bias across studies (see Item 15). | Figure 1  8-10 |
| Additional analysis | 23 | Give results of additional analyses, if done (e.g., sensitivity or subgroup analyses, meta-regression [see Item 16]). | Figure 1B and efigure 4. |
| DISCUSSION | | | |
| Summary of evidence | 24 | Summarize the main findings including the strength of evidence for each main outcome; consider their relevance to key groups (e.g., healthcare providers, users, and policy makers). | 14 |
| Limitations | 25 | Discuss limitations at study and outcome level (e.g., risk of bias), and at review-level (e.g., incomplete retrieval of identified research, reporting bias). | 14-16 |
| Conclusions | 26 | Provide a general interpretation of the results in the context of other evidence, and implications for future research. | 16-20 |
| FUNDING | | | |
| Funding | 27 | Describe sources of funding for the systematic review and other support (e.g., supply of data); role of funders for the systematic review. | 21 |

**eAppendix 1: methods**

**METHODS**

For the meta-review of non-CNS dysfunction in first episode psychosis (FEP), we selected meta-analyses reporting markers of immune, cardiometabolic and HPA system differences between patient and control groups, rather than differences in rates of diagnoses of conditions based on pre-defined diagnostic criteria^1-4^ (e.g. rates of diagnoses of type 2 diabetes mellitus or hypercholesterolemia). The rationale behind this methodology was threefold. Firstly, patients with psychotic illness are less likely to seek medical attention and so there is the risk of under-reporting of diagnoses^5^. Secondly, certain conditions such as glucose and lipid dysregulation develop on a continuum and take time for serum/plasma markers to reach threshold for a diagnosis. For example, changes in glucose regulation occur 4-7 years prior to diagnosis of diabetes^6^. Thirdly, physiological alterations that do not meet diagnostic thresholds can, nevertheless, be associated with worsened mortality/morbidity outcomes. For example, there is robust evidence that low-grade inflammation is an independent risk factor for atherosclerosis and cardiovascular disease^7^. In the meta-review of CNS dysfunction in FEP, we aimed to summarise evidence for CNS alterations considered reliably implicated in FEP for comparison with the non-CNS alterations. In view of this, our search focussed on parameters identified in an expert review as key aspects of the neurobiology of schizophrenia^8^. These were brain structural (assessed using volumetric magnetic resonance imaging (MRI) and diffusion tensor imaging), neurophysiological (assessed using electroencephalography and functional MRI) and neurochemical (assessed using magnetic resonance spectroscopy) disturbances. This was not intended to be exhaustive, but to be representative of CNS alterations established as implicated in schizophrenia. Our search was limited to FEP, and where possible, antipsychotic naïve individuals, as with the meta-review of non-CNS alterations.

**Search Strategy**

Two systematic meta-reviews, one for CNS alterations and the other for non-CNS alterations, were performed according to Preferred Reporting Items for Systematic Reviews and Meta-Analyses (PRISMA)^9^ (Supplementary Information, eTable 1). Two reviewers (T.P. and E.D.) independently searched Pubmed from 1990 to May week 2 2017 for each systematic meta-review. For non-CNS parameters, three organ systems recognised to exhibit dysregulation in schizophrenia were focussed upon: the immune, cardiometabolic and HPA systems. As such, the following search terms were used: meta-analysis and (psychosis or schizophr*) and (immune OR inflamm* OR cytokine OR antibody OR cardiac OR metabolic OR glucose OR diabetes OR lipid OR cholesterol OR triglyceride OR antioxidant OR cortisol OR hypothalamic pituitary adrenal OR HPA OR prolactin). For CNS parameters, search terms were guided by outcomes from an expert review^8^ that specifies recognised neurobiological anomalies in schizophrenia. As such, the following search terms were used: meta-analysis and (psychosis or schizophr*) and (brain volume or gray matter or grey matter or VBM or white matter tract* or fMRI or BOLD or lateral* or DTI or P300 or P50 or pre-pulse or mismatch negativity or pursuit or N-acetyl aspartate or REM or phosphomonoester or dopamine or dendrite or gliosis or NMDA). 1990 was identified as an appropriate date from which to commence our searches, since few research syntheses were published prior to that year^10,11^. Searches were complemented by hand searching of reference lists from review articles.

The patient inclusion and exclusion criteria for each meta-review were the same to permit comparison of the outcome measures. For non-CNS parameters, inclusion criteria were: 1) Study design: meta-analyses of observational studies comparing patient groups with a healthy control groups; 2) Patients to meet criteria for a first episode of psychosis^12^; 3) Peripheral measurements of biochemical or hematological disturbances in patients and controls (e.g. serum, plasma, or saliva). Specific parameters included were as follows: immune disturbance: cytokine and lymphocyte counts; cardio-metabolic disturbance: glucose, insulin, insulin resistance, cholesterol, triglyceride and anti-oxidant levels; HPA axis disturbance: baseline (morning) cortisol, cortisol awakening response, and prolactin levels. Studies in any language were considered. Exclusion criteria were: 1) meta-analyses that included patients with multiple episodes of psychosis; 2) meta-analyses that included patients with substance or medication induced psychotic disorder; 3) meta-analyses that reported on dichotomous presence/absence of raised parameters as defined by diagnostic criteria^1-4^ (e.g. rates of diagnoses of type 2 diabetes mellitus).

For CNS parameters, inclusion criteria were: 1) Study design: meta-analyses of observational studies comparing patient groups with a healthy control groups; 2) Patients to meet criteria for a first episode of psychosis. Specific parameters included were as follows: structural measures (e.g. brain volume and fractional isotropy analyses); electroencephalographic measures (e.g. mismatch negativity and P300 analyses), and neurochemical parameters (e.g. N-acetyl aspartate levels). Exclusion criteria were: 1) Meta-analyses that included patients with multiple episodes of psychosis; 2) Meta-analyses that included patients with substance or medication induced psychotic disorder 3) Meta-analyses that did not provide effect sizes as a synthesis of their findings.

Search processes for both CNS and non-CNS alterations are documented in eFigures 1 and 2 respectively.

To statistically compare the magnitude of effect sizes between different CNS and non-CNS systems in FEP, all meta-analyses were repeated. Patient and control data from the studies referenced in the meta-analyses our search terms had identified were extracted independently by T.P. and E.D. according to the following model: author, year of publication, country, design, whether patient groups were antipsychotic naive, number of patients/controls, and mean (SD) measure of immune / cardiometabolic / HPA / brain structural / neurophsysiological / neurochemical parameter. Data were only extracted for those CNS and non-CNS parameters where there were significant differences demonstrated between FEP and controls in the original meta-analyses. A 2-tailed *P* < 0.05 was deemed significant for all statistical analyses. A minimum of 3 studies were required to run a meta-analysis.

**Assessment of methodological quality**

Methodological quality of selected meta-analyses was assessed independently by two authors (R.M. and E.D.) using the AMSTAR (‘A Measurement Tool to Assess Systematic Reviews’) checklist (eAppendix). Since T.P. and O.D. are authors of meta-analyses revealed by the search terms, they were excluded from the AMSTAR assessments to avoid bias. The AMSTAR is a tool that consists of 11 questions assessing study quality. Scores of 8 to 11 were deemed to be of high quality, 4 to 7 of medium quality, and 0 to 3 of low quality^13^. Meta-analyses that scored lower than 4 out of 11 were excluded, as were studies that failed to use appropriate methods to combine studies (question 9 of the AMSTAR) (eTables 2 and 3, aggregate scores documented in eTables 4 and 5). Heterogeneity scores for samples within each meta-analysis were also recorded, as assessed using the χ^2^ test, Q or I^2^ statistic (eTables 2 and 3). An I^2^ of less than 25% was deemed to have low heterogeneity, 25-75% medium heterogeneity, and greater than 75% high heterogeneity^14^.


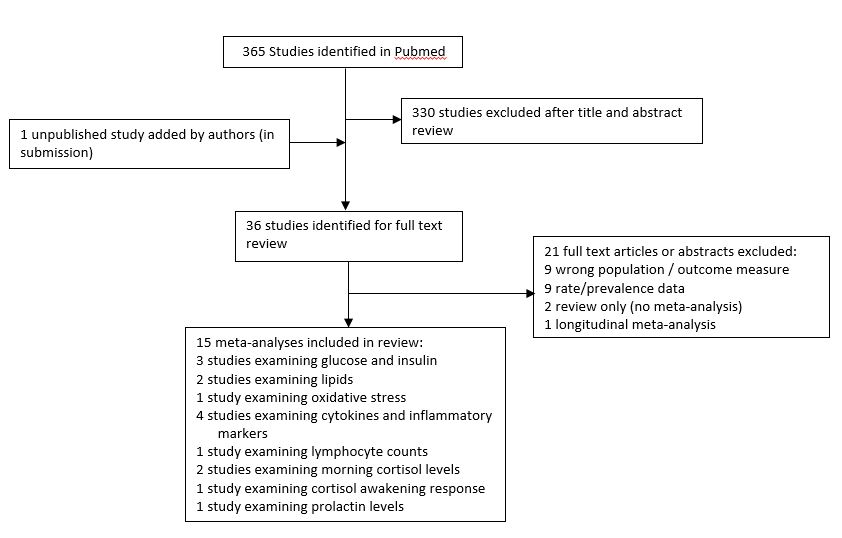


**eFigure 1: Search process for meta-analyses examining non-CNS disturbances in First Episode Psychosis and individuals at Clinical High Risk for psychosis.**


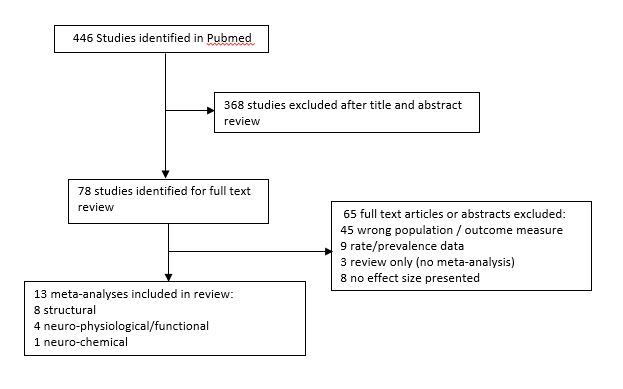


**eFigure 2: Search process for meta-analyses examining CNS disturbances in First Episode Psychosis and individuals at Clinical High Risk for psychosis.**

**eTable 2: AMSTAR Methodological Quality results for meta-analyses examining non-CNS dysfunction in FEP/CHR individuals.**

|  | **1** | **2** | **3** | **4** | **5** | **6** | **7** | **8** | **9** | **10** | **11** |
| --- | --- | --- | --- | --- | --- | --- | --- | --- | --- | --- | --- |
| Pillinger et al., 2017^15^ | Y | Y | Y | N | Y | Y | Y | Y | Y | Y | Y |
| Perry et al., 2016^16^ | Y | Y | Y | N | Y | Y | Y | Y | Y | Y | Y |
| Greenhalgh et al., 2016^17^ | Y | Y | Y | N | Y | N | Y | Y | Y | Y | Y |
| Misiak et al., 2017^18^ | Y | Y | Y | Y | Y | Y | Y | Y | Y | Y | Y |
| Pillinger et al., 2017^19^ | Y | Y | Y | N | Y | Y | Y | Y | Y | Y | Y |
| Flatow et al., 2013^20^ | Y | Y | Y | N | N | N | Y | Y | Y | N | Y |
| Upthegrove et al., 2014^21^ | Y | Y | Y | N | Y | Y | N | N | Y | N | Y |
| Goldsmith et al., 2016^22^ | Y | Y | Y | N | Y | N | Y | Y | Y | Y | Y |
| Miller et al., 2011^23^ | Y | Y | Y | N | N | N | Y | Y | Y | N | Y |
| Fernandes et al., 2016^24^ | Y | Y | Y | N | Y | N | Y | Y | Y | Y | Y |
| Miller et al., 2013^25^ | Y | Y | Y | N | N | N | Y | Y | Y | N | Y |
| Berger et al., 2016^26^ | Y | Y | Y | Y | Y | Y | Y | Y | Y | N | Y |
| Chaumette et al., 2016^27^ | Y | Y | Y | N | Y | Y | Y | Y | Y | N | Y |
| Girshkin et al., 2014^28^ | Y | Y | Y | N | Y | Y | Y | Y | Y | Y | Y |
| Gonzalez-Blanco et al., 2016^29^ | Y | Y | Y | N | Y | N | Y | Y | Y | Y | Y |

**eTable 3: AMSTAR Methodological Quality results for meta-analyses examining non-CNS dysfunction in FEP/CHR individuals.**

|  | **1** | **2** | **3** | **4** | **5** | **6** | **7** | **8** | **9** | **10** | **11** |
| --- | --- | --- | --- | --- | --- | --- | --- | --- | --- | --- | --- |
| Adriano et al., 2012^30^ | Y | N | Y | N | Y | Y | N | N | Y | Y | Y |
| Walter et al., 2016^31^ | Y | N | Y | N | N | Y | Y | N | Y | Y | Y |
| Adriano et al., 2010^32^ | Y | N | Y | N | N | Y | N | N | Y | Y | Y |
| Haijma et al., 2013^33^ | Y | N | N | N | N | Y | N | N | Y | Y | Y |
| Vita and de Peri, 2007^34^ | Y | N | Y | N | N | N | N | N | Y | N | Y |
| de Peri et al., 2012^35^ | Y | N | Y | N | N | Y | N | N | Y | Y | Y |
| Vita et al., 2006^36^ | Y | N | Y | N | N | Y | N | N | Y | N | Y |
| Fusar-Poli et al., 2012^37^ | Y | N | Y | N | N | N | N | N | Y | Y | Y |
| Erickson et al., 2016^38^ | Y | N | Y | N | N | N | N | N | Y | Y | Y |
| Qiu et al., 2014^39^ | Y | Y | Y | N | Y | Y | Y | N | Y | Y | Y |
| Chen et al., 2014^40^ | Y | N | Y | N | N | N | N | N | N | Y | Y |
| Haigh et al., 2017^41^ | Y | N | Y | N | N | N | N | N | Y | N | Y |
| Brugger et al., 2011^42^ | Y | N | Y | N | N | N | N | N | Y | Y | Y |

Abbreviations: N: no; Y: yes.

**eTable 4: Rosenthal’s Fail Safe N scores for non-CNS and CNS parameters included in FEP final analysis**

|  | Parameter | Fail Safe N |
| --- | --- | --- |
| IMMUNE | IL-1β | 426 |
|  | sIL-2R | 26 |
|  | IL-6 | 1639 |
|  | TNF-α | 872 |
|  | TGF-β | 37 |
|  | CRP | 217 |
|  | Lymphocyte count | 17 |
| CARDIO-METABOLIC | Fasting glucose | 43 |
|  | Glucose post-OGTT | 56 |
|  | Fasting insulin | 97 |
|  | Insulin resistance | 76 |
|  | Triglycerides | 26 |
|  | Total cholesterol | 64 |
|  | LDL cholesterol | 60 |
| HPA | Prolactin | 125 |
|  | CAR | 75 |
| STRUCTURAL | Total brain volume | 268 |
|  | Total gray matter | 128 |
|  | R hippocampal volume | 592 |
|  | L hippocamal volume | 663 |
|  | Thalamus volume | 83 |
|  | RCN volume | 27 |
|  | LCN volume | 66 |
|  | Total CSF | 50 |
|  | R lateral ventricle | 64 |
|  | L lateral ventricle | 111 |
| PHYSIOLOGICAL | Auditory P300 amplitude | 1100 |
|  | Duration-deviant MMN | 450 |
| CHEMICAL | Thalamus NAA | 19 |
|  | Frontal cortex NAA | 359 |
|  | Temporal cortex NAA | 35 |

**eFigure 3 Forest plot for magnitude of immune, cardiometabolic, HPA, brain structural, neurophysiological, and neurochemical alterations in antipsychotic naïve first episode psychosis compared with healthy controls.** Each line represents a summary effect size for a meta-analysis in one parameter: squares represent the summary effect size for that parameter, with the horizontal line running through each square illustrating the width of the overall 95% CI. Blue diamonds represent summary effect sizes for immune, cardiometabolic, HPA, structural, neurophysiological, and neurochemical systems: the middle of each diamond represents the summary effect size, and the width of the diamond depicts the width of the overall 95% CI. Red diamonds represent summary effect sizes and accompanying 95% CI for non-CNS and CNS effect sizes. ES: effect size; CNS: central nervous system; FEP: first episode psychosis; HPA: hypothalamic pituitary adrenal axis; IL1β: interleukin-1β; sIL2-R: soluble interleukin-2 receptor; IL6: interleukin-6; TGFβ: transforming growth factor-β; CRP: C-reactive protein; NAA: N-acetylaspartic acid; N: number.

**
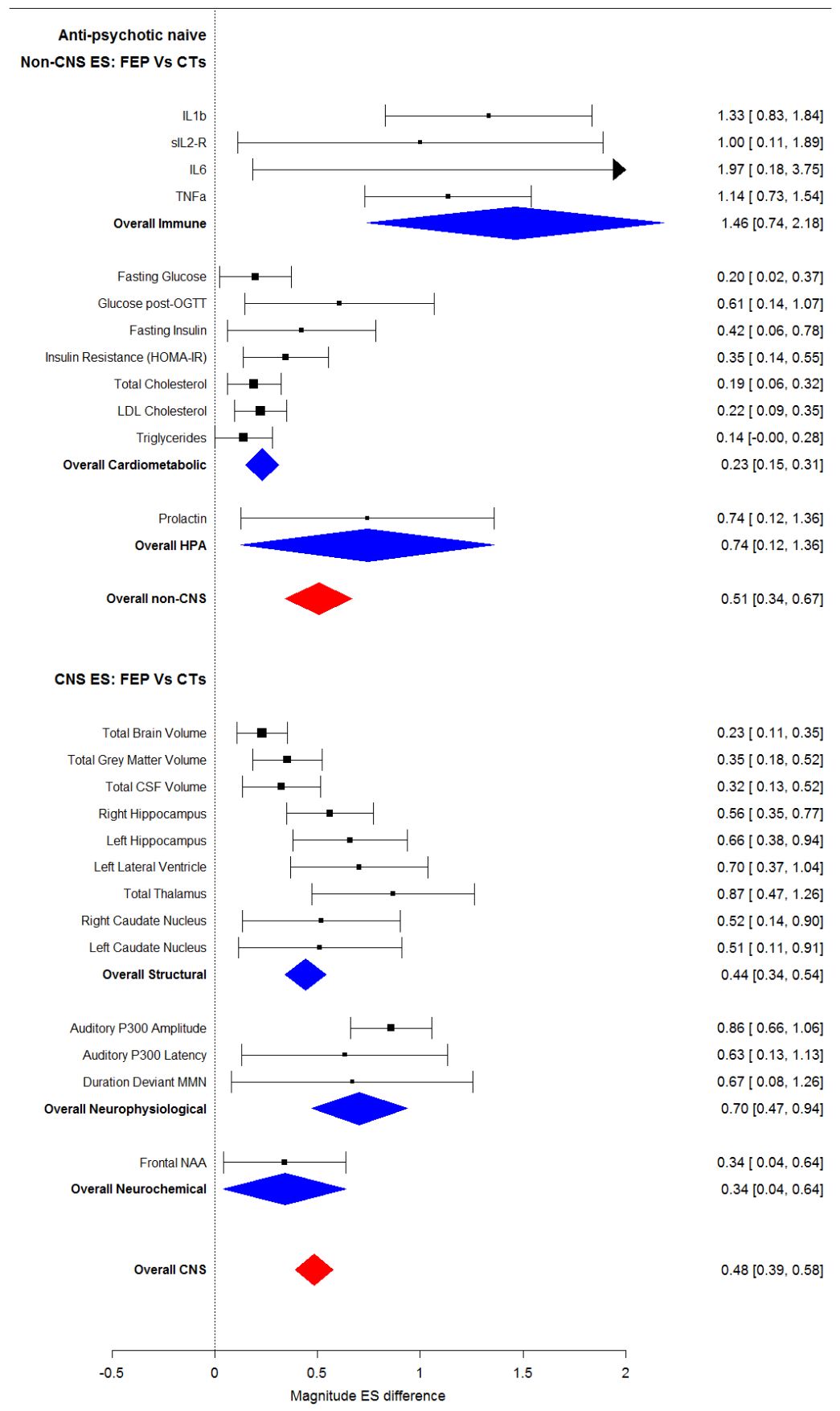
**

**eFigure 4 Figure 2: Heat map comparing relative magnitude of effect sizes (ES) for immune, hypothalamic pituitary adrenal (HPA) axis, cardiometabolic, brain structural, neurophysiological, and neurochemical alterations in antipsychotic naïve first episode psychosis.** The map is read from left to right, comparing parameters on the y axis with parameters on the x axis. A negative Wald score (blue squares) demonstrates that the parameter ES on the y axis is numerically lower compared with the intersecting parameter ES on the x axis. A positive Wald score (red squares) demonstrates that the parameter ES on the y axis is numerically higher than the intersecting parameter ES on the x axis. Numbers within the squares are the P values that accompany the Wald score.

**
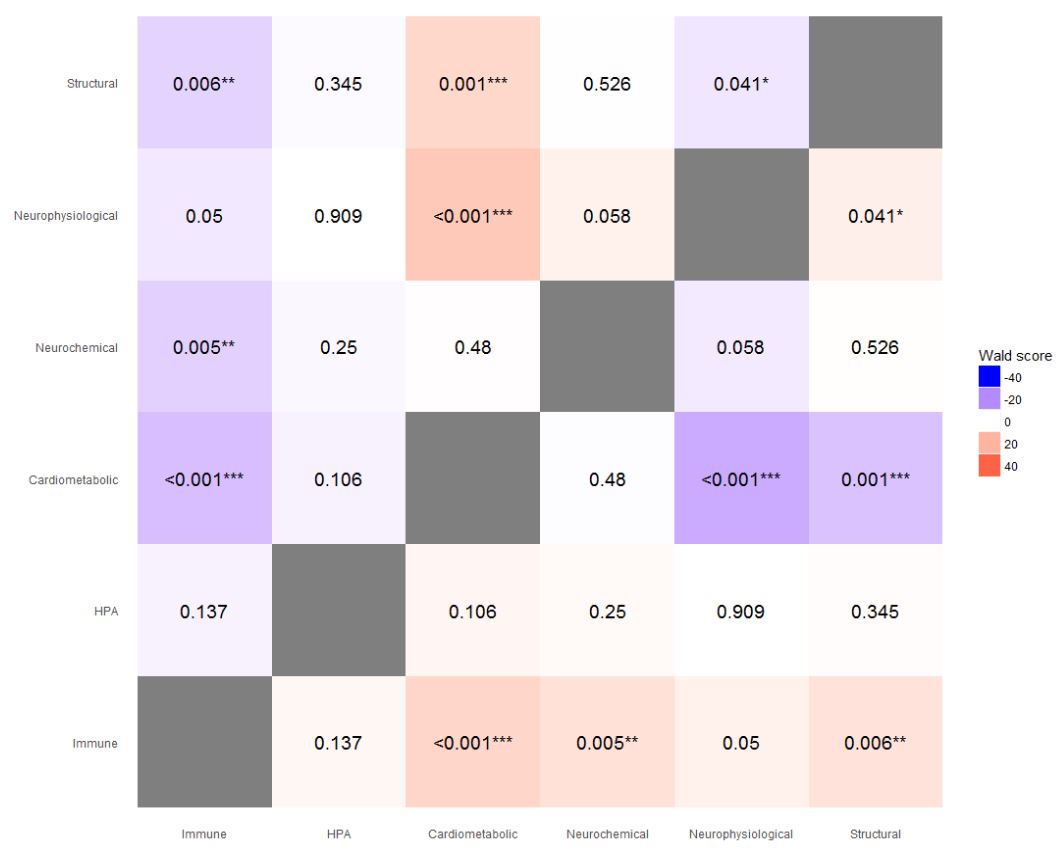
**

**eAppendix 2**

**AMSTAR Checklist**

**1. Was an 'a priori' design provided?**

**2. Was there duplicate study selection and data extraction?**

**3. Was a comprehensive literature search performed?**

**4. Was the status of publication (i.e. grey literature) used as an inclusion criterion?**

**5. Was a list of studies (included and excluded) provided?**

**6. Were the characteristics of the included studies provided?**

**7. Was the scientific quality of the included studies assessed and documented?**

**8. Was the scientific quality of the included studies used appropriately in formulating conclusions?**

**9. Were the methods used to combine the findings of studies appropriate?**

**10. Was the likelihood of publication bias assessed?**

**11. Was the conflict of interest included?**

**eAppendix 3**

**Case control studies included in meta- and Wald analyses**

Aas M, Dazzan P, Mondelli V, Toulopoulou T, Reichenberg A, Di Forti M et al. Abnormal cortisol awakening response predicts worse cognitive function in patients with first-episode psychosis. Psychological Medicine 2011; 41(3): 463-476.

Akiyama K. Serum levels of soluble IL-2 receptor alpha, IL-6 and IL-1 receptor antagonist in schizophrenia before and during neuroleptic administration. Schizophrenia Research 1999; 37(1): 97-106.

Albayrak Y, Beyazyuz M, Beyazyuz E, Kuloglu M. Increased serum prolactin levels in drug-naive first-episode male patients with schizophrenia. Nord J Psychiatry 2014; 68(5): 341-346.

Angelopoulos EK, Markianos M, Daskalopoulou EG, Hatzimanolis J, Tzemos J. Changes in central serotonergic function as a correlate of duration of illness in paranoid schizophrenia. Psychiatry Research 2002; 110(1): 9-17.

Arranz B, Rosel P, Ramirez N, Duenas R, Fernandez P, Sanchez JM et al. Insulin resistance and increased leptin concentrations in noncompliant schizophrenia patients but not in antipsychotic-naive first-episode schizophrenia patients. J Clin Psychiat 2004; 65(10): 1335-1342.

Atkinson RJ, Michie PT, Schall U. Duration Mismatch Negativity and P3a in First-Episode Psychosis and Individuals at Ultra-High Risk of Psychosis. Biol Psychiat 2012; 71(2): 98-104.

Bartha R, Al-Semaan YM, Williamson PC, Drost DJ, Malla AK, Carr TJ et al. A short echo proton magnetic resonance spectroscopy study of the left mesial-temporal lobe in first-onset schizophrenic patients. Biol Psychiat 1999; 45(11): 1403-1411.

Basoglu C, Cetin M, Omer O, Ebrinc S, Baser U, Kandilcioglu H et al. Comparison of right thalamus and temporal cortex metabolite levels of drug-naive first-episode psychotic and chronic schizophrenia in patients. Turk Psikiyatri Dergisi 2006; 17(2): 85-91.

Basoglu C, Oner O, Gunes C, Semiz UB, Ates AM, Algul A et al. Plasma orexin A, ghrelin, cholecystokinin, visfatin, leptin and agouti-related protein levels during 6-week olanzapine treatment in first-episode male patients with psychosis. International Clinical Psychopharmacology 2010; 25(3): 165-171.

Berge D, Carmona S, Rovira M, Bulbena A, Salgado P, Vilarroya O. Gray matter volume deficits and correlation with insight and negative symptoms in first-psychotic-episode subjects. Acta Psychiatr Scand 2011; 123(6): 431-439.

Bertolino A, Callicott JH, Mattay VS, Weidenhammer KM, Rakow R, Egan MF et al. The effect of treatment with antipsychotic drugs on brain N-acetylaspartate measures in patients with schizophrenia. Biol Psychiat 2001; 49(1): 39-46.

Bodatsch M, Ruhrmann S, Wagner M, Muller R, Schultze-Lutter F, Frommann I et al. Prediction of psychosis by mismatch negativity. Biol Psychiatry 2011; 69(10): 959-966.

Boonstra G, van Haren NEM, Schnack HG, Cahn W, Burger H, Boersma M et al. Brain Volume Changes After Withdrawal of Atypical Antipsychotics in Patients With First-Episode Schizophrenia. J Clin Psychopharm 2011; 31(2): 146-153.

Borovcanin M, Jovanovic I, Radosavljevic G, Dejanovic SD, Bankovic D, Arsenijevic N et al. Elevated serum level of type-2 cytokine and low IL-17 in first episode psychosis and schizophrenia in relapse. Journal of Psychiatric Research 2012; 46(11): 1421-1426.

Brown KJ, Gonsalvez CJ, Harris AW, Williams LM, Gordon E. Target and non-target ERP disturbances in first episode vs. chronic schizophrenia. Clin Neurophysiol 2002; 113(11): 1754-1763.

Bustillo JR, Lauriello J, Rowland LM, Thomson LM, Petropoulos H, Hammond R et al. Longitudinal follow-up of neurochemical changes during the first year of antipsychotic treatment in schizophrenia patients with minimal previous medication exposure. Schizophrenia Research 2002; 58(2-3): 313-321.

Bustillo JR, Rowland LM, Jung R, Brooks WM, Qualls C, Hammond R et al. Proton magnetic resonance spectroscopy during initial treatment with antipsychotic medication in schizophrenia. Neuropsychopharmacology 2008; 33(10): 2456-2466.

Cahn W, Hulshoff Pol HE, Bongers M, Schnack HG, Mandl RC, Van Haren NE et al. Brain morphology in antipsychotic-naive schizophrenia: a study of multiple brain structures. Br J Psychiatry Suppl 2002; 43: s66-72.

Cecil KM, Lenkinski RE, Gur RE, Gur RC. Proton magnetic resonance spectroscopy in the frontal and temporal lobes of neuroleptic naive patients with schizophrenia. Neuropsychopharmacology 1999; 20(2): 131-140.

Chakos MH, Schobel SA, Gu H, Gerig G, Bradford D, Charles C et al. Duration of illness and treatment effects on hippocampal volume in male patients with schizophrenia. Br J Psychiatry 2005; 186: 26-31.

Chen BW, H.X.; Zhang, M.D. P300 characteristics in patients with first-episode schizophrenia and its follow-up study. Shan-Xi Yike Daxue Xuebao 2010; 41: 253-256.

Chen DC, Du XD, Yin GZ, Yang KB, Nie Y, Wang N et al. Impaired glucose tolerance in first-episode drug-naive patients with schizophrenia: relationships with clinical phenotypes and cognitive deficits. Psychological Medicine 2016; 46(15): 3219-3230.

Chen S, Broqueres-You D, Yang GG, Wang ZR, Li YL, Yang FD et al. Male sex may be associated with higher metabolic risk in first-episode schizophrenia patients: A preliminary study. Asian Journal of Psychiatry 2016; 21: 25-30.

Chen XS, Lu YZ, Wang JJ, Wang HX, Zhang MD, Lou FY et al. Relationship between event-related potential P300 and first episode schizophrenia. Chinese Med J-Peking 2007; 120(4): 339-341.

Chua SE, Cheung C, Cheung V, Tsang JT, Chen EY, Wong JC et al. Cerebral grey, white matter and csf in never-medicated, first-episode schizophrenia. Schizophr Res 2007; 89(1-3): 12-21.

Chua SE, Lam IWS, Tai KS, Cheung C, Tang WN, Chen EYH et al. Brain morphological abnormality in schizophrenia is independent of country of origin. Acta Psychiat Scand 2003; 108(4): 269-275.

Cohn TA, Remington G, Zipursky RB, Azad A, Connolly P, Wolever TMS. Insulin resistance and adiponectin levels in drug-free patients with schizophrenia: A preliminary report (vol 51, pg 382, 2006). Can J Psychiat 2006; 51(8): 552-552.

Coscia DM, Narr KL, Robinson DG, Hamilton LS, Sevy S, Burdick KE et al. Volumetric and shape analysis of the thalamus in first-episode schizophrenia. Hum Brain Mapp 2009; 30(4): 1236-1245.

Crespo-Facorro B, Kim J, Andreasen NC, O'Leary DS, Magnotta V. Regional frontal abnormalities in schizophrenia: a quantitative gray matter volume and cortical surface size study. Biol Psychiatry 2000; 48(2): 110-119.

Crespo-Facorro B, Roiz-Santianez R, Perez-Iglesias R, Tordesillas-Gutierrez D, Mata I, Rodriguez-Sanchez JM et al. Specific brain structural abnormalities in first-episode schizophrenia. A comparative study with patients with schizophreniform disorder, non-schizophrenic non-affective psychoses and healthy volunteers. Schizophr Res 2009; 115(2-3): 191-201.

Dasgupta A, Singh OP, Rout JK, Saha T, Mandal S. Insulin resistance and metabolic profile in antipsychotic naive schizophrenia patients. Progress in Neuro-Psychopharmacology & Biological Psychiatry 2010; 34(7): 1202-1207.

Davatzikos C, Shen D, Gur RC, Wu X, Liu D, Fan Y et al. Whole-brain morphometric study of schizophrenia revealing a spatially complex set of focal abnormalities. Arch Gen Psychiatry 2005; 62(11): 1218-1227.

De Berardis D, Conti CM, Marini S, Serroni N, Moschetta FS, Carano A et al. C-Reactive Protein Level and Its Relationship with Suicide Risk and Alexithymia among Newly Diagnosed, Drug-Naive Patients with Non-Affective Psychosis. Eur J Inflamm 2013; 11(1): 215-221.

Degreef G, Ashtari M, Bogerts B, Bilder RM, Jody DN, Alvir JM et al. Volumes of ventricular system subdivisions measured from magnetic resonance images in first-episode schizophrenic patients. Arch Gen Psychiatry 1992; 49(7): 531-537.

DeLisi LE, Hoff AL, Schwartz JE, Shields GW, Halthore SN, Gupta SM et al. Brain morphology in first-episode schizophrenic-like psychotic patients: a quantitative magnetic resonance imaging study. Biol Psychiatry 1991; 29(2): 159-175.

Demiralp T, Ucok A, Devrim M, Isoglu-Alkac U, Tecer A, Polich J. N2 and P3 components of event-related potential in first-episode schizophrenic patients: scalp topography, medication, and latency effects. Psychiatry Res 2002; 111(2-3): 167-179.

Devrim-Ucok M, Keskin-Ergen HY, Ucok A. Novelty P3 and P3b in first-episode schizophrenia and chronic schizophrenia. Prog Neuropsychopharmacol Biol Psychiatry 2006; 30(8): 1426-1434.

Di Nicola M, Cattaneo A, Hepgul N, Di Forti M, Aitchison KJ, Janiri L et al. Serum and gene expression profile of cytokines in first-episode psychosis. Brain Behav Immun 2013; 31: 90-95.

Ding ML, Song XQ, Zhao JY, Gao JS, Li X, Yang G et al. Activation of Th17 cells in drug naive, first episode schizophrenia. Progress in Neuro-Psychopharmacology & Biological Psychiatry 2014; 51: 78-82.

Ebdrup BH, Glenthoj B, Rasmussen H, Aggernaes B, Langkilde AR, Paulson OB et al. Hippocampal and caudate volume reductions in antipsychotic-naive first-episode schizophrenia. J Psychiatry Neurosci 2010; 35(2): 95-104.

Enez Darcin A, Yalcin Cavus S, Dilbaz N, Kaya H, Dogan E. Metabolic syndrome in drug-naive and drug-free patients with schizophrenia and in their siblings. Schizophr Res 2015; 166(1-3): 201-206.

Falcone T, Carlton E, Lee C, Janigro M, Fazio V, Forcen FE et al. Does Systemic Inflammation Play a Role in Pediatric Psychosis? Clin Schizophr Relat Psychoses 2015; 9(2): 65-78b.

Fannon D, Chitnis X, Doku V, Tennakoon L, O'Ceallaigh S, Soni W et al. Features of structural brain abnormality detected in first-episode psychosis. Am J Psychiat 2000; 157(11): 1829-1834.

Fannon D, Simmons A, Tennakoon L, O'Ceallaigh S, Sumich A, Doku V et al. Selective deficit of hippocampal N-acetylaspartate in antipsychotic-naive patients with schizophrenia. Biol Psychiat 2003; 54(6): 587-598.

Fawzi MH, Fawzi MM, Fawzi MM, Said NS. C-reactive protein serum level in drug-free male Egyptian patients with schizophrenia. Psychiatry Research 2011; 190(1): 91-97.

Fernandez-Egea E, Bernardo M, Donner T, Conget I, Parellada E, Justicia A et al. Metabolic profile of antipsychotic-naive individuals with non-affective psychosis. Brit J Psychiat 2009; 194(5): 434-438.

Galinska B, Szulc A, Tarasow E, Kubas B, Dzienis W, Czernikiewicz A et al. Duration of untreated psychosis and proton magnetic resonance spectroscopy (1H-MRS) findings in first-episode schizophrenia. Med Sci Monit 2009; 15(2): CR82-88.

Ganguli R, Rabin BS. Increased Serum Interleukin-2 Receptor Concentration in Schizophrenic and Brain-Damaged Subjects. Archives of General Psychiatry 1989; 46(3): 292-293.

Garcia-Rizo C, Fernandez-Egea E, Oliveira C, Justicia A, Parellada E, Bernardo M et al. Prolactin concentrations in newly diagnosed, antipsychotic-naive patients with nonaffective psychosis. Schizophr Res 2012; 134(1): 16-19.

Garcia-Rizo C, Kirkpatrick B, Fernandez-Egea E, Oliveira C, Bernardo M. Abnormal glycemic homeostasis at the onset of serious mental illnesses: A common pathway. Psychoneuroendocrinology 2016; 67: 70-75.

Gattaz WF, Dalgalarrondo P, Schroder HC. Abnormalities in Serum Concentrations of Interleukin-2, Interferon-Alpha and Interferon-Gamma in Schizophrenia Not Detected. Schizophrenia Research 1992; 6(3): 237-241.

Gilbert AR, Rosenberg DR, Harenski K, Spencer S, Sweeney JA, Keshavan MS. Thalamic volumes in patients with first-episode schizophrenia. Am J Psychiatry 2001; 158(4): 618-624.

Glenthoj A, Glenthoj BY, Mackeprang T, Pagsberg AK, Hemmingsen RP, Jernigan TL et al. Basal ganglia volumes in drug-naive first-episode schizophrenia patients before and after short-tenn treatment with either a typical or an atypical antipsychotic drug. Psychiat Res-Neuroim 2007; 154(3): 199-208.

Hepgul N, Pariante CM, Dipasquale S, DiForti M, Taylor H, Marques TR et al. Childhood maltreatment is associated with increased body mass index and increased C-reactive protein levels in first-episode psychosis patients. Psychological Medicine 2012; 42(9): 1893-1901.

Hermens DF, Ward PB, Hodge MA, Kaur M, Naismith SL, Hickie IB. Impaired MMN/P3a complex in first-episode psychosis: cognitive and psychosocial associations. Prog Neuropsychopharmacol Biol Psychiatry 2010; 34(6): 822-829.

Higuchi Y, Seo T, Miyanishi T, Kawasaki Y, Suzuki M, Sumiyoshi T. Mismatch negativity and p3a/reorienting complex in subjects with schizophrenia or at-risk mental state. Front Behav Neurosci 2014; 8: 172.

Higuchi Y, Sumiyoshi T, Seo T, Miyanishi T, Kawasaki Y, Suzuki M. Mismatch Negativity and Cognitive Performance for the Prediction of Psychosis in Subjects with At-Risk Mental State. Plos One 2013; 8(1).

Hirayasu Y, Asato N, Ohta H, Hokama H, Arakaki H, Ogura C. Abnormalities of auditory event-related potentials in schizophrenia prior to treatment. Biol Psychiatry 1998; 43(4): 244-253.

Hsieh MH, Shan JC, Huang WL, Cheng WC, Chiu MJ, Jaw FS et al. Auditory event-related potential of subjects with suspected pre-psychotic state and first-episode psychosis. Schizophrenia Research 2012; 140(1-3): 243-249.

Ichimiya T, Okubo Y, Suhara T, Sudo Y. Reduced volume of the cerebellar vermis in neuroleptic-naive schizophrenia. Biol Psychiat 2001; 49(1): 20-27.

James ACD, Crow TJ, Renowden S, Wardell AMJ, Smith DM, Anslow P. Is the course of brain development in schizophrenia delayed? Evidence from onsets in adolescence. Schizophrenia Research 1999; 40(1): 1-10.

Jayakumar PN, Venkatasubramanian G, Gangadhar BN, Janakiramaiah N, Keshavan MS. Optimized voxel-based morphometry of gray matter volume in first-episode, antipsychotic-naive schizophrenia. Prog Neuropsychopharmacol Biol Psychiatry 2005; 29(4): 587-591.

John JP, Burgess PW, Yashavantha BS, Shakeel MK, Halahalli HN, Jain S. Differential relationship of frontal pole and whole brain volumetric measures with age in neuroleptic-naive schizophrenia and healthy subjects. Schizophr Res 2009; 109(1-3): 148-158.

Kalmady SV, Venkatasubramanian G, Shivakumar V, Gautham S, Subramaniam A, Jose DA et al. Relationship between Interleukin-6 Gene Polymorphism and Hippocampal Volume in Antipsychotic-Naive Schizophrenia: Evidence for Differential Susceptibility? Plos One 2014; 9(5).

Kaur M, Battisti RA, Lagopoulos J, Ward PB, Hickie IB, Hermens DF. Neurophysiological biomarkers support bipolar-spectrum disorders within psychosis cluster. J Psychiatr Neurosci 2012; 37(5): 313-321.

Kaur M, Battisti RA, Ward PB, Ahmed A, Hickie IB, Hermens DF. MMN/P3a deficits in first episode psychosis: Comparing schizophrenia-spectrum and affective-spectrum subgroups. Schizophrenia Research 2011; 130(1-3): 203-209.

Kavzoglu SO, Hariri AG. Intracellular Adhesion Molecule (ICAM-1), Vascular Cell Adhesion Molecule (VCAM-1) and E-Selectin Levels in First Episode Schizophrenic Patients. Klin Psikofarmakol B 2013; 23(3): 205-214.

Keshavan MS, Rosenberg D, Sweeney JA, Pettegrew JW. Decreased caudate volume in neuroleptic-naive psychotic patients. Am J Psychiatry 1998; 155(6): 774-778.

Kim YK, Kim L, Lee MS. Relationships between interleukins, neurotransmitters and psychopathology in drug-free male schizophrenics. Schizophrenia Research 2000; 44(3): 165-175.

Kim YK, Myint AM, Lee BH, Han CS, Lee HJ, Kim DJ et al. Th1, Th2 and Th3 cytokine alteration in schizophrenia. Progress in Neuro-Psychopharmacology & Biological Psychiatry 2004; 28(7): 1129-1134.

Kim YK, Myint AM, Verkerk R, Scharpe S, Steinbusch H, Leonard B. Cytokine Changes and Tryptophan Metabolites in Medication-Naive and Medication-Free Schizophrenic Patients. Neuropsychobiology 2009; 59(2): 123-129.

Kirkpatrick B, Garcia-Rizo C, Tang K, Fernandez-Egea E, Bernardo M. Cholesterol and triglycerides in antipsychotic-naive patients with nonaffective psychosis. Psychiatry Research 2010; 178(3): 559-561.

Korostenskaja M, Dapsys K, Siurkute A, Maciulis V, Ruksenas O, Kahkonen S. Effects of risperidone on auditory information processing in neuroleptic-naive patients with schizophrenia spectrum disorders. Acta Neurobiol Exp (Wars) 2006; 66(2): 139-144.

Kubistova A, Horacek J, Novak T. Increased interleukin-6 and tumor necrosis factor alpha in first episode schizophrenia patients versus healthy controls. Psychiatr Danub 2012; 24 Suppl 1: S153-156.

Laakso MP, Tiihonen J, Syvalahti E, Vilkman H, Laakso A, Alakare B et al. A morphometric MRI study of the hippocampus in first-episode, neuroleptic-naive schizophrenia. Schizophr Res 2001; 50(1-2): 3-7.

Lacerda ALT, Hardan AY, Yorbik O, Vemulapalli M, Prasad KM, Keshavan MS. Morphology of the orbitofrontal cortex in first-episode schizophrenia: Relationship with negative symptomatology. Progress in Neuro-Psychopharmacology & Biological Psychiatry 2007; 31(2): 510-516.

Lawrie SM, Whalley HC, Abukmeil SS, Kestelman JN, Donnelly L, Miller P et al. Brain structure, genetic liability, and psychotic symptoms in subjects at high risk of developing schizophrenia. Biol Psychiat 2001; 49(10): 811-823.

Lee SY, Namkoong K, Cho HH, Song DH, An SK. Reduced visual P300 amplitudes in individuals at ultra-high risk for psychosis and first-episode schizophrenia. Neuroscience Letters 2010; 486(3): 156-160.

Liu S, Sun N, Xu Y, Yang CX, Ren Y, Liu ZF et al. Possible Association of the GSK3 beta Gene with the Anxiety Symptoms of Major Depressive Disorder and P300 Waveform. Genet Test Mol Bioma 2012; 16(12): 1382-1389.

Magno E, Yeap S, Thakore JH, Garavan H, De Sanctis P, Foxe JJ. Are auditory-evoked frequency and duration mismatch negativity deficits endophenotypic for schizophrenia? High-density electrical mapping in clinically unaffected first-degree relatives and first-episode and chronic schizophrenia. Biol Psychiatry 2008; 64(5): 385-391.

Masserini C, Vita A, Basile R, Morselli R, Boato P, Peruzzi C et al. Lymphocyte Subsets in Schizophrenic Disorders - Relationship with Clinical, Neuromorphological and Treatment Variables. Schizophrenia Research 1990; 3(4): 269-275.

Matsumoto H, Simmons A, Williams S, Pipe R, Murray R, Frangou S. Structural magnetic imaging of the hippocampus in early onset schizophrenia. Biol Psychiat 2001; 49(10): 824-831.

Meisenzahl E, Seifert D, Bottlender R, Teipel S, Zetzsche T, Jager M et al. Differences in hippocampal volume between major depression and schizophrenia: a comparative neuroimaging study. European Archives of Psychiatry and Clinical Neuroscience 2010; 260(2): 127-137.

Misiak B, Laczmanski L, Sloka NK, Szmida E, Piotrowski P, Loska O et al. Metabolic dysregulation in first-episode schizophrenia patients with respect to genetic variation in one-carbon metabolism. Psychiatry Research 2016; 238: 60-67.

Miyaoka T, Yasukawa R, Mizuno S, Sukegawa T, Inagaki T, Horiguchi J et al. Proton magnetic resonance spectroscopy (1H-MRS) of hippocampus, basal ganglia, and vermis of cerebellum in schizophrenia associated with idiopathic unconjugated hyperbilirubinemia (Gilbert's syndrome). J Psychiatr Res 2005; 39(1): 29-34.

Molina V, Sanz J, Sarramea F, Benito C, Palomo T. Lower prefrontal gray matter volume in schizophrenia in chronic but not in first episode schizophrenia patients. Psychiatry Res 2004; 131(1): 45-56.

Molina V, Sanz J, Sarramea F, Luque R, Benito C, Palomo T. No association between dorsolateral prefrontal gray matter deficit and N-acetyl aspartate ratios in schizophrenia. Neuropsychobiology 2006; 54(3): 171-178.

Mondelli V, Ciufolini S, Murri MB, Bonaccorso S, Di Forti M, Giordano A et al. Cortisol and Inflammatory Biomarkers Predict Poor Treatment Response in First Episode Psychosis. Schizophrenia Bull 2015; 41(5): 1162-1170.

Mondelli V, Dazzan P, Hepgul N, Di Forti M, Aas M, D'Albenzio A et al. Abnormal cortisol levels during the day and cortisol awakening response in first-episode psychosis: The role of stress and of antipsychotic treatment. Schizophrenia Research 2010; 116(2-3): 234-242.

Mondragon-Maya A, Solis-Vivanco R, Leon-Ortiz P, Rodriguez-Agudelo Y, Yanez-Tellez G, Bernal-Hernandez J et al. Reduced P3a amplitudes in antipsychotic naive first-episode psychosis patients and individuals at clinical high-risk for psychosis. Journal of Psychiatric Research 2013; 47(6): 755-761.

Narr KL, Thompson PM, Szeszko P, Robinson D, Jang S, Woods RP et al. Regional specificity of hippocampal volume reductions in first-episode schizophrenia. Neuroimage 2004; 21(4): 1563-1575.

Niemann K, Hammers A, Coenen VA, Thron A, Klosterkotter J. Evidence of a smaller left hippocampus and left temporal horn in both patients with first episode schizophrenia and normal control subjects. Psychiat Res-Neuroim 2000; 99(2): 93-110.

Nopoulos P, Torres I, Flaum M, Andreasen NC, Ehrhardt JC, Yuh WT. Brain morphology in first-episode schizophrenia. Am J Psychiatry 1995; 152(12): 1721-1723.

Oades RD, Wild-Wall N, Juran SA, Sachsse J, Oknina LB, Ropcke B. Auditory change detection in schizophrenia: sources of activity, related neuropsychological function and symptoms in patients with a first episode in adolescence, and patients 14 years after an adolescent illness-onset. BMC Psychiatry 2006; 6: 7.

Ohrmann P, Kugel H, Bauer J, Siegmund A, Kolkebeck K, Suslow T et al. Learning potential on the WCST in schizophrenia is related to the neuronal integrity of the anterior cingulate cortex as measured by proton magnetic resonance spectroscopy. Schizophr Res 2008; 106(2-3): 156-163.

Ohrmann P, Siegmund A, Suslow T, Pedersen A, Spitzberg K, Kersting A et al. Cognitive impairment and in vivo metabolites in first-episode neuroleptic-naive and chronic medicated schizophrenic patients: a proton magnetic resonance spectroscopy study. J Psychiatr Res 2007; 41(8): 625-634.

Ohrmann P, Siegmund A, Suslow T, Spitzberg K, Kersting A, Arolt V et al. Evidence for glutamatergic neuronal dysfunction in the prefrontal cortex in chronic but not in first-episode patients with schizophrenia: a proton magnetic resonance spectroscopy study. Schizophr Res 2005; 73(2-3): 153-157.

Olbrich HM, Valerius G, Rusch N, Buchert M, Thiel T, Hennig J et al. Frontolimbic glutamate alterations in first episode schizophrenia: evidence from a magnetic resonance spectroscopy study. World J Biol Psychiatry 2008; 9(1): 59-63.

O'Neill J, Levitt J, Caplan R, Asarnow R, McCracken JT, Toga AW et al. 1H MRSI evidence of metabolic abnormalities in childhood-onset schizophrenia. Neuroimage 2004; 21(4): 1781-1789.

Ozgurdal S, Gudlowski Y, Witthaus H, Kawohl W, Uhl I, Hauser M et al. Reduction of auditory event-related P300 amplitude in subjects with at-risk mental state for schizophrenia. Schizophrenia Research 2008; 105(1-3): 272-278.

Petrikis P, Tigas S, Tzallas AT, Papadopoulos I, Skapinakis P, Mavreas V. Parameters of glucose and lipid metabolism at the fasted state in drug-naive first-episode patients with psychosis: Evidence for insulin resistance. Psychiatry Research 2015; 229(3): 901-904.

Premkumar P, Kumari V, Corr PJJ, Sharma T. Frontal lobe volumes in schizophrenia: Effects of stage and duration of illness. Journal of Psychiatric Research 2006; 40(7): 627-637.

Preuss UW, Zetzsche T, Jager M, Groll C, Frodl T, Bottlender R et al. Thalamic volume in first-episode and chronic schizophrenic subjects: a volumetric MRI study. Schizophrenia Research 2005; 73(1): 91-101.

Pruessner M, Lepage M, Collins DL, Pruessner JC, Joober R, Malla AK. Reduced hippocampal volume and hypothalamus-pituitary-adrenal axis function in first episode psychosis: evidence for sex differences. Neuroimage Clin 2015; 7: 195-202.

Puri BK, Hutton SB, Saeed N, Oatridge A, Hajnal JV, Duncan LJ et al. A serial longitudinal quantitative MRI study of cerebral changes in first-episode schizophrenia using image segmentation and subvoxel registration. Psychiat Res-Neuroim 2001; 106(2): 141-150.

Rao ML, Gross G, Strebel B, Braunig P, Huber G, Klosterkotter J. Serum amino acids, central monoamines, and hormones in drug-naive, drug-free, and neuroleptic-treated schizophrenic patients and healthy subjects. Psychiatry Res 1990; 34(3): 243-257.

Rapaport MH, Lohr JB. Serum-Soluble Interleukin-2 Receptors in Neuroleptic-Naive Schizophrenic Subjects and in Medicated Schizophrenic Subjects with and without Tardive-Dyskinesia. Acta Psychiat Scand 1994; 90(5): 311-315.

Razi K, Greene KP, Sakuma M, Ge SM, Kushner M, DeLisi LE. Reduction of the parahippocampal gyrus and the hippocampus in patients with chronic schizophrenia. Brit J Psychiat 1999; 174: 512-519.

Rizos EN, Papathanasiou M, Michalopoulou PG, Mazioti A, Douzenis A, Kastania A et al. Association of serum BDNF levels with hippocampal volumes in first psychotic episode drug-naive schizophrenic patients. Schizophr Res 2011; 129(2-3): 201-204.

Rosa PGP, Schaufelberger MS, Uchida RR, Duran FLS, Lappin JM, Menezes PR et al. Lateral ventricle differences between first-episode schizophrenia and first-episode psychotic bipolar disorder: A population-based morphometric MRI study. World Journal of Biological Psychiatry 2010; 11(7): 873-887.

Ryan MCM, Collins P, Thakore JH. Impaired fasting glucose tolerance in first-episode, drug-naive patients with schizophrenia. Am J Psychiat 2003; 160(2): 284-289.

Saddichha S, Manjunatha N, Ameen S, Akhtar S. Diabetes and schizophrenia - effect of disease or drug? Results from a randomized, double-blind, controlled prospective study in first-episode schizophrenia. Acta Psychiat Scand 2008; 117(5): 342-347.

Saddichha S, Manjunatha N, Ameen S, Akhtar S. Metabolic syndrome in first episode schizophrenia - A randomized double-blind controlled, short-term prospective study (vol 101, pg 266, 2008). Schizophrenia Research 2008; 104(1-3): 307-308.

Salgado-Pineda P, Baeza I, Perez-Gomez M, Vendrell P, Junque C, Bargallo N et al. Sustained attention impairment correlates to gray matter decreases in first episode neuroleptic-naive schizophrenic patients. Neuroimage 2003; 19(2 Pt 1): 365-375.

Salisbury DF, Polizzotto NR, Nestor PG, Haigh SM, Koehler J, McCarley RW. Pitch and Duration Mismatch Negativity and Premorbid Intellect in the First Hospitalized Schizophrenia Spectrum. Schizophrenia Bull 2017; 43(2): 407-416.

Salisbury DF, Shenton ME, Sherwood AR, Fischer IA, Yurgelun-Todd DA, Tohen M et al. First-episode schizophrenic psychosis differs from first-episode affective psychosis and controls in P300 amplitude over left temporal lobe. Arch Gen Psychiatry 1998; 55(2): 173-180.

Salokangas RKR, Cannon T, Van Erp T, Ilonen T, Taiminen T, Karlsson H et al. Structural magnetic resonance imaging in patients with first-episode schizophrenia, psychotic and severe non-psychotic depression and healthy controls - Results of the Schizophrenia and Affective Psychoses (SAP) project. Brit J Psychiat 2002; 181: S58-S65.

Sarandol A, Sarandol E, Acikgoz HE, Eker SS, Akkaya C, Dirican M. First-episode psychosis is associated with oxidative stress: Effects of short-term antipsychotic treatment. Psychiatry and Clinical Neurosciences 2015; 69(11): 699-707.

Scherk H, Backens M, Zill P, Schneider-Axmann T, Wobrock T, Usher J et al. SNAP-25 genotype influences NAA/Cho in left hippocampus. J Neural Transm (Vienna) 2008; 115(11): 1513-1518.

Sengupta S, Parrilla-Escobar MA, Klink R, Fathalli F, Ng YK, Stip E et al. Are metabolic indices different between drug-naive first-episode psychosis patients and healthy controls? Schizophrenia Research 2008; 102(1-3): 329-336.

Shrivastava A, Tamhane M. Serum prolactin level and severity of psychopathology in patients of schizophrenia. Indian J Psychiatry 2000; 42(1): 48-51.

Sirota P, Meiman M, Herschko R, Bessler H. Effect of neuroleptic administration on serum levels of soluble IL-2 receptor-alpha and IL-1 receptor antagonist in schizophrenic patients. Psychiatry Research 2005; 134(2): 151-159.

Smith GN, Lang DJ, Kopala LC, Lapointe JS, Falkai P, Honer WG. Developmental abnormalities of the hippocampus in first-episode schizophrenia. Biol Psychiat 2003; 53(7): 555-561.

Solis-Vivanco R, Mondragon-Maya A, Leon-Ortiz P, Rodriguez-Agudelo Y, Cadenhead KS, de la Fuente-Sandoval C. Mismatch Negativity reduction in the left cortical regions in first-episode psychosis and in individuals at ultra high-risk for psychosis. Schizophrenia Research 2014; 158(1-3): 58-63.

Song X, Fan X, Zhang J, Zheng H, Li X, Pang L et al. Prolactin serum levels correlate with inflammatory status in drug-naive first-episode schizophrenia. World J Biol Psychiatry 2014; 15(7): 546-552.

Song XQ, Fan XD, Song XW, Zhang JJ, Zhang W, Li X et al. Elevated levels of adiponectin and other cytokines in drug naive, first episode schizophrenia patients with normal weight. Schizophrenia Research 2013; 150(1): 269-273.

Song XQ, Lv LX, Li WQ, Hao YH, Zhao JP. The Interaction of Nuclear Factor-Kappa B and Cytokines Is Associated with Schizophrenia. Biol Psychiat 2009; 65(6): 481-488.

Spelman LM, Walsh PI, Sharifi N, Collins P, Thakore JH. Impaired glucose tolerance in first-episode drug-naive patients with schizophrenia. Diabetic Medicine 2007; 24(5): 481-485.

Sperner-Unterweger B, Whitworth A, Kemmler G, Hilbe W, Thaler J, Weiss G et al. T-cell subsets in schizophrenia: a comparison between drug-naive first episode patients and chronic schizophrenic patients. Schizophrenia Research 1999; 38(1): 61-70.

Srihari VH, Phutane VH, Ozkan B, Chwastiak L, Ratliff JC, Woods SW et al. Cardiovascular mortality in schizophrenia: Defining a critical period for prevention. Schizophrenia Research 2013; 146(1-3): 64-68.

Stanley JA, Vemulapalli M, Nutche J, Montrose DM, Sweeney JA, Pettegrew JW et al. Reduced N-acetyl-aspartate levels in schizophrenia patients with a younger onset age: a single-voxel 1H spectroscopy study. Schizophr Res 2007; 93(1-3): 23-32.

Sumich A, Chitnis XA, Fannon DG, O'Ceallaigh S, Doku VC, Falrowicz A et al. Temporal lobe abnormalities in first-episode psychosis. Am J Psychiat 2002; 159(7): 1232-1234.

Sun HQ, Li SX, Chen FB, Zhang Y, Li P, Jin M et al. Diurnal neurobiological alterations after exposure to clozapine in first-episode schizophrenia patients. Psychoneuroendocrinology 2016; 64: 108-116.

Szeszko PR, Goldberg E, Gunduz-Bruce H, Ashtari M, Robinson D, Malhotra AK et al. Smaller anterior hippocampal formation volume in antipsychotic-naive patients with first-episode schizophrenia. Am J Psychiatry 2003; 160(12): 2190-2197.

Takahashi T, Wood SJ, Soulsby B, McGorry PD, Tanino R, Suzuki M et al. Follow-up MRI study of the insular cortex in first-episode psychosis and chronic schizophrenia. Schizophr Res 2009; 108(1-3): 49-56.

Theberge J, Bartha R, Drost DJ, Menon RS, Malla A, Takhar J et al. Glutamate and glutamine measured with 4.0 T proton MRS in never-treated patients with schizophrenia and healthy volunteers. Am J Psychiatry 2002; 159(11): 1944-1946.

Theodoropoulou S, Spanakos G, Baxevanis CN, Economou M, Gritzapis AD, Papamichail MP et al. Cytokine serum levels, autologous mixed lymphocyte reaction and surface marker analysis in never medicated and chronically medicated schizophrenic patients. Schizophrenia Research 2001; 47(1): 13-25.

Thomas MA, Ke Y, Levitt J, Caplan R, Curran J, Asarnow R et al. Preliminary study of frontal lobe 1H MR spectroscopy in childhood-onset schizophrenia. J Magn Reson Imaging 1998; 8(4): 841-846.

Umbricht DS, Bates JA, Lieberman JA, Kane JM, Javitt DC. Electrophysiological indices of automatic and controlled auditory information processing in first-episode, recent-onset and chronic schizophrenia. Biol Psychiatry 2006; 59(8): 762-772.

van der Stelt O, Lieberman JA, Belger A. Auditory P300 in high-risk, recent-onset and chronic schizophrenia. Schizophr Res 2005; 77(2-3): 309-320.

Velakoulis D, Pantelis C, McGorry PD, Dudgeon P, Brewer W, Cook M et al. Hippocampal volume in first-episode psychoses and chronic schizophrenia: a high-resolution magnetic resonance imaging study. Arch Gen Psychiatry 1999; 56(2): 133-141.

Venkatasubramanian G, Chittiprol S, Neelakantachar N, Naveen MN, Thirthall J, Gangadhar BN et al. Insulin and insulin-like growth factor-1 abnormalities in anti psychotic-naive schizophrenia. Am J Psychiat 2007; 164(10): 1557-1560.

Venkatasubramanian G, Chittiprol S, Neelakantachar N, Shetty TK, Gangadhar BN. A Longitudinal Study on the Impact of Antipsychotic Treatment on Serum Leptin in Schizophrenia. Clinical Neuropharmacology 2010; 33(6): 288-292.

Venkatasubramanian G, Gangadhar BN, Jayakumar PN, Keshavan MS. Striato-cerebellar abnormalities in never-treated schizophrenia. Biol Psychiat 2003; 53(8): 23s-23s.

Venkatasubramanian G, Jayakumar PN, Gangadhar BN, Keshavan MS. Automated MRI parcellation study of regional volume and thickness of prefrontal cortex (PFC) in antipsychotic-naive schizophrenia. Acta Psychiatr Scand 2008; 117(6): 420-431.

Venkatraman TN, Hamer RM, Perkins DO, Song AW, Lieberman JA, Steen RG. Single-voxel 1H PRESS at 4.0 T: precision and variability of measurements in anterior cingulate and hippocampus. NMR Biomed 2006; 19(4): 484-491.

Verma S, Sitoh YY, Ho YC, Poon LY, Subramaniam M, Chan YH et al. Hippocampal volumes in first-episode psychosis. J Neuropsychiatry Clin Neurosci 2009; 21(1): 24-29.

Verma SK, Subramaniam M, Liew A, Poon LY. Metabolic Risk Factors in Drug-Naive Patients With First-Episode Psychosis. J Clin Psychiat 2009; 70(7): 997-1000.

Wang CHL, Y.; Su, L.Y.; Ma, J.D., Mu, J.L. Effects of olanzapine and risperidone on memory function and P300 in patients with first episode schizophrenia. Zhongguo Shenjing Jingshen Jibing Zazhi 2009; 35: 481-484.

Wang J, Hirayasu Y, Hiramatsu K, Hokama H, Miyazato H, Ogura C. Increased rate of P300 latency prolongation with age in drug-naive and first episode schizophrenia. Clin Neurophysiol 2003; 114(11): 2029-2035.

Wang J, Tang Y, Li C, Mecklinger A, Xiao Z, Zhang M et al. Decreased P300 current source density in drug-naive first episode schizophrenics revealed by high density recording. Int J Psychophysiol 2010; 75(3): 249-257.

Wang QS, X.L.; Ma, X.H.; Shi, J.J.; Yao, J. et al. Event-related potentials and molecular genetics in patients with first-episode schizophrenia and their relatives. Zhongguo Shenjing Jingshen Jibing Zazhi 2004; 30: 229-230.

Wani RA, Dar MA, Margoob MA, Haq YHRI, Haq I, Shah MS. Diabetes mellitus and impaired glucose tolerance in patients with schizophrenia, before and after antipsychotic treatment. J Neurosci Rural Pra 2015; 6(1): 17-22.

Whitworth AB, Honeder M, Kremser C, Kemmler G, Felber S, Hausmann A et al. Hippocampal volume reduction in male schizophrenic patients. Schizophr Res 1998; 31(2-3): 73-81.

Whitworth AB, Kemmler G, Honeder M, Kremser C, Felber S, Hausmann A et al. Longitudinal volumetric MRI study in first- and multiple-episode male schizophrenia patients. Psychiatry Res 2005; 140(3): 225-237.

Witthaus H, Mendes U, Brune M, Ozgurdal S, Bohner G, Gudlowski Y et al. Hippocampal subdivision and amygdalar volumes in patients in an at-risk mental state for schizophrenia. J Psychiatr Neurosci 2010; 35(1): 33-40.

Wood SJ, Berger GE, Wellard RM, Proffitt T, McConchie M, Velakoulis D et al. A 1H-MRS investigation of the medial temporal lobe in antipsychotic-naive and early-treated first episode psychosis. Schizophr Res 2008; 102(1-3): 163-170.

Wood SJ, Velakoulis D, Smith DJ, Bond D, Stuart GW, McGorry PD et al. A longitudinal study of hippocampal volume in first episode psychosis and chronic schizophrenia. Schizophr Res 2001; 52(1-2): 37-46.

Wu XL, Huang ZP, Wu RR, Zhong ZY, Wei QL, Wang HL et al. The comparison of glycometabolism parameters and lipid profiles between drug-naive, first-episode schizophrenia patients and healthy controls. Schizophrenia Research 2013; 150(1): 157-162.

Yasukawa R, Miyaoka T, Mizuno S, Inagaki T, Horiguchi J, Oda K et al. Proton magnetic resonance spectroscopy of the anterior cingulate gyrus, insular cortex and thalamus in schizophrenia associated with idiopathic unconjugated hyperbilirubinemia (Gilbert's syndrome). J Psychiatr Neurosci 2005; 30(6): 416-422.

Zabala A, Sanchez-Gonzalez J, Parellada M, Moreno DM, Reig S, Burdalo MT et al. Findings of proton magnetic resonance spectometry in the dorsolateral prefrontal cortex in adolescents with first episodes of psychosis. Psychiatry Res 2007; 156(1): 33-42.

Zhang XY, Chen DC, Tan YL, An HM, Zunta-Soares GB, Huang XF et al. Glucose disturbances in first-episode drug-naive schizophrenia: Relationship to psychopathology. Psychoneuroendocrinology 2015; 62: 376-380.

Zipursky RB, Lambe EK, Kapur S, Mikulis DJ. Cerebral gray matter volume deficits in first episode psychosis. Arch Gen Psychiatry 1998; 55(6): 540-546.

**eAppendix 4:**

**Proportion of sample with a DSM diagnosis of schizophrenia (non-CNS studies)**

**DNA: data not available; SCZ: schizophrenia**

| **Author** | **Year** | **%SCZ** |
| --- | --- | --- |
| Nicola | 2012 | DNA |
| Song | 2009 | 100 |
| Theodoropoulou | 2001 | 100 |
| Kim | 2000 | 100 |
| Song | 2013 | 100 |
| Rapoport | 1994 | 100 |
| Falcone | 2015 | DNA |
| Akiyama | 1999 | 100 |
| Gataz | 1992 | 100 |
| Sirota | 2005 | 100 |
| Borovcanin | 2012 | DNA |
| Ganguli | 1994 | 100 |
| Ding | 2014 | 100 |
| Kalmady | 2014 | 100 |
| Kubistova | 2012 | 100 |
| Kim | 2009 | 100 |
| Kim | 2004 | 100 |
| Hepgul | 2012 | DNA |
| Fawzi | 2011 | 100 |
| Berardis | 2013 | 83 |
| Masserini | 1990 | 100 |
| Sperner Unterweger | 1999 | 100 |
| Zhang | 2015 | 100 |
| Petrikis | 2015 | DNA |
| Ryan | 2003 | 100 |
| Venkatasubramanian | 2007 | 100 |
| Cohn | 2006 | 100 |
| Spelman | 2007 | 100 |
| Wani | 2015 | 100 |
| Saddichha | 2008 | 100 |
| Garcia Rizo | 2016 | 41 |
| Enez Darcin | 2015 | 100 |
| Sun | 2016 | 100 |
| Fernandez Egea | 2009 | 70 |
| Dasgupta | 2010 | 100 |
| Arranz | 2004 | 100 |
| Chen, Du et al | 2016 | 100 |
| Chen, Broqueres-You | 2016 | 100 |
| Kirkpatrick | 2010 | DNA |
| Wu | 2013 | 100 |
| Srihari | 2013 | 100 |
| Venkatasubramanian | 2010 | 100 |
| Misiak | 2016 | 100 |
| Sarandol | 2015 | DNA |
| Kavzoglu | 2013 | 100 |
| Sengupta | 2008 | 42 |
| Verma | 2009 | DNA |
| Basoglu | 2010 | 100 |
| Aas | 2011 | DNA |
| Mondelli | 2015 | DNA |
| Pruessner | 2015 | DNA |
| Albayrak | 2013 | 100 |
| Angelopoulos | 2002 | 100 |
| Rao | 1990 | 100 |
| Shrivastava | 2000 | 100 |
| Song | 2014 | 100 |
| **TOTAL** |  | **74%** |

**Proportion of sample with a DSM diagnosis of schizophrenia (CNS studies)**

**DNA: data not available; SCZ: schizophrenia**

| **Author** | **Year** | **%SCZ** |
| --- | --- | --- |
| Berge | 2011 | DNA |
| Cahn | 2002 | 100 |
| Chua | 2007 | 100 |
| Ebdrup | 2010 | 100 |
| Ichimiya | 2001 | 100 |
| John | 2009 | 100 |
| Lacerda | 2007 | 100 |
| Rizos | 2011 | 100 |
| Nopoulos | 1995 | 100 |
| James | 1999 | 100 |
| Fannon | 2000 | DNA |
| Lawrie | 2001 | 100 |
| Matsumoto | 2001 | 100 |
| Chua | 2003 | 100 |
| Premkumar | 2006 | 100 |
| Takahashi | 2009 | 70 |
| Rosa | 2010 | 100 |
| Witthaus | 2010 | 100 |
| Boonstra | 2011 | 100 |
| Whitworth | 1998 | 100 |
| Velakoulis | 1999 | DNA |
| Davatzikos | 2005 | 100 |
| Jayakumar | 2005 | 100 |
| Molina | 2005 | 100 |
| Salgado-Pineda | 2003 | 100 |
| Venkatasubramanian | 2008 | 100 |
| Zipursky | 1998 | 100 |
| Laakso | 2001 | 100 |
| Narr male | 2004 | 100 |
| Narr female | 2004 | 100 |
| Molina | 2005 | 100 |
| Szesko | 2003 | 100 |
| Chakos | 2005 | 100 |
| Meisenzahl | 2009 | 100 |
| Niemann | 2000 | 100 |
| Razi | 1999 | 100 |
| Smith | 2003 | 100 |
| Sumich | 2002 | DNA |
| Verma | 2009 | DNA |
| Whitworth | 2005 | 100 |
| Wood | 2001 | DNA |
| Degreef | 1992 | 100 |
| Puri | 2001 | 100 |
| Salokangas | 2002 | 100 |
| Delisi | 1991 | DNA |
| Salokangas | 2002 | 100 |
| Coscia female | 2009 | 100 |
| Coscia male | 2009 | 100 |
| Gilbert | 2001 | 100 |
| Crespo-Facorro | 2009 | 100 |
| Venkatasubramanian | 2003 | 100 |
| Glenthoj | 2007 | 100 |
| Keshevan | 1998 | 77 |
| Brown | 2002 | 100 |
| Chen | 2010 | 100 |
| Chen | 2007 | 100 |
| Demirapl | 2002 | 100 |
| Devrim-ucok | 2006 | 100 |
| Korostenskaja | 2006 | DNA |
| Lee | 2010 | 0 |
| Ozgurdal | 2008 | 100 |
| Salisbury | 1998 | 100 |
| Umbricht | 2006 | 100 |
| van-der Stelt | 2005 | 100 |
| Wang | 2009 | 100 |
| Wang | 2003 | 100 |
| Wang | 2010 | 100 |
| Wang | 2004 | 100 |
| Oades | 2006 | 100 |
| Hermens | 2010 | 6 |
| Bodatsch | 2011 | 100 |
| Kaur | 2011 | DNA |
| Kaur | 2012 | DNA |
| Atkinson | 2012 | DNA |
| Atkinson | 2012 | DNA |
| Hsieh | 2012 | DNA |
| Higuchi | 2013 | 100 |
| Higuchi | 2014 | 100 |
| Mondragon-Maya | 2013 | DNA |
| Solis-Vivanco | 2014 | DNA |
| Salisbury | 2017 | DNA |
| O'Neill | 2004 | 100 |
| Theberge | 2002 | 100 |
| Yasukawa | 2005 | 100 |
| Yasukawa | 2005 | 100 |
| Bustillo | 2002 | 100 |
| Bustillo | 2008 | 100 |
| Cecil | 1999 | 100 |
| Fannon | 2003 | 100 |
| Ohrmann | 2005 | 100 |
| Ohrmann | 2007 | 100 |
| Ohrmann | 2008 | 100 |
| Ohrmann | 2008 | 100 |
| Olbrich | 2008 | 100 |
| Stanley | 2007 | 100 |
| Stanley | 2007 | 100 |
| Thomas | 1998 | 100 |
| Venkatraman | 2006 | 100 |
| Zabala | 2007 | 50 |
| Bartha | 1999 | 100 |
| Basoglu | 2006 | DNA |
| Bertolino | 2001 | 100 |
| Galinska | 2009 | 100 |
| Miyaoka | 2005 | 100 |
| Scherk | 2008 | 100 |
| Wood | 2008 | 100 |
| Magno | 2008 | 100 |
| **TOTAL:** |  | **84%** |

**eBox 1: Summary of strengths and weaknesses of the putative models linking CNS and non-CNS alterations in psychosis**

| **Strengths and weaknesses of the potential models linking CNS and non-CNS alterations in psychosis** |
| --- |
| **Model 1: non-CNS dysfunction impacts CNS function, increasing psychosis risk**  Strengths:   - Supported by evidence of rare cases where non-CNS dysfunction leads to psychosis. - Population based cohort studies suggest that raised inflammatory markers in childhood are risk factors for later development of psychosis. - Hypercortisolemia, inflammation, and oxidative stress are risk factors for conversion to FEP in the prodrome.   Weaknesses:   - Many associations between non-CNS dysfunction and psychosis have not shown evidence of causation to date. To demonstrate causality, non-CNS dysfunction needs to be addressed prior to the onset of psychosis, for example in the prodrome, and show that the development of psychosis is prevented. - Explains rare cases but is unlikely to account for typical cases of schizophrenia where CNS alterations are thought to occur early in neurodevelopment unless non-CNS alterations occur very early in development.   **Model 2: non-CNS dysfunction emerges as a consequence of psychosis**  Strengths:   - Resolution of acute psychosis is associated with normalisation of previously elevated cytokines (IL-1β, IL-6, and TGF-β). - Some non-CNS alterations are not seen in the prodrome and develop later in the illness, suggesting they are a consequence of psychosis or its treatment.   Weaknesses:   - Population based studies indicate that some alterations precede psychosis. - Reduction in levels of cytokines with the resolution of an acute psychotic episode could be part of the therapeutic action of treatment (supportive of model 1 rather than 2).   **Model 3: a shared risk factor plays a role in development of psychosis and non-CNS alterations, potentially through divergent mechanisms**  Strengths:   - Heterogeneity in both non-CNS and CNS findings between patients suggests divergent mechanisms underlie them. - Lack of consistent relationships between a number of non-CNS and CNS alterations   Weaknesses:   - Certain correlations e.g. between glucose dysregulation, insulin resistance and PANSS positive scores in FEP may point towards these non-CNS alterations being linked with the clinical expression of psychosis, suggestive of a common pathoetiological mechanism, in a least a subgroup of non-CNS alterations. - In general, there is a paucity of studies testing relationships between non-CNS and CNS alterations |

**eAppendix 5**

**Protocol:**

Valid at time of literature search (week 2 May 2017)

Working title: Meta-review of CNS and non-CNS alterations in first episode psychosis

Language: English

**Type of review:**

Systematic review and meta-analysis

**Language:**

English

**Details of any existing review of the same topic by the same authors:**

None

**Named Contact:**

Dr Toby Pillinger

Department of Psychosis Studies

Institute of Psychiatry, Psychology and Neuroscience

16 de Crespigny Park

Camberwell

SE5 8AF

United Kingdom

Toby.pillinger@kcl.ac.uk

**Collaborators:**

Dr Toby Pillinger ([toby.pillinger@kcl.ac.uk](mailto:toby.pillinger@kcl.ac.uk))

Dr Enrico D’Ambrosio ([enrico.dambrosio@kcl.ac.uk](mailto:enrico.dambrosio@kcl.ac.uk))

Dr Robert McCutcheon (Robert.mccutcheon@kcl.ac.uk)

Professor Oliver Howes ([oliver.howes@kcl.ac.uk](mailto:oliver.howes@kcl.ac.uk))

**Institution:**

Institute of Psychiatry, Psychology and Neuroscience, King’s College London

**Funding Sources:**

MRC-UK, Maudsley Charity, Brain&Behavior Research Foundation, Wellcome Trust.

**Conflicts of Interest:**

Dr Howes has received investigator-initiated research funding from and/or participated in advisory/ speaker meetings organised by Astra-Zeneca, Autifony, BMS, Eli Lilly, Heptares, Janssen, Lundbeck, Lyden-Delta, Otsuka, Servier, Sunovion, Rand and Roche. Neither Professor Howes nor his family have been employed by or have holdings/a financial stake in any biomedical company. Drs Pillinger, Beck and Stubbs report no financial relationships with commercial interests.

**Review Question:**

People with schizophrenia and related psychotic disorders show abnormalities in several organ systems in addition to the central nervous system (CNS); and this contributes to excess mortality. However, it is unclear how strong the evidence is for alterations across multiple non-CNS systems at the onset of psychosis, how the alterations in non-CNS systems compare to findings in the CNS, or how they relate to symptoms.

**Searches:**

Database: Pubmed

Non-CNS review search terms: meta-analysis and (psychosis or schizophr*) and (immune OR inflamm* OR cytokine OR antibody OR cardiac OR metabolic OR glucose OR diabetes OR lipid OR cholesterol OR triglyceride OR antioxidant OR cortisol OR hypothalamic pituitary adrenal OR HPA OR prolactin).

CNS search terms: meta-analysis and (psychosis or schizophr*) and (brain volume or gray matter or grey matter or VBM or white matter tract* or fMRI or BOLD or lateral* or DTI or P300 or P50 or pre-pulse or mismatch negativity or pursuit or N-acetyl aspartate or REM or phosphomonoester or dopamine or dendrite or gliosis or NMDA).

**Condition being studied:**

Alterations in immune, cardiometabolic, HPA, brain structural, neurophysiological, and neurochemical parameters.

**Participants/Population, and Comparator/Control:**

First episode psychosis, healthy controls.

**Primary Outcomes:**

Effect sizes of CNS and non-CNS parameter alterations in FEP compared with healthy controls

Statistical comparison of CNS and non-CNS summary effect sizes

**Data Extraction:**

Screening based on title and abstract will be performed independently by two authors (T.P. and E.D.).

Data extraction from case-control studies referenced in selected meta-analyses will be performed independently (by T.P. and E.D.), and any disagreements resolved by rechecking original articles.

**Risk of Bias Assessment:**

Orwin’s fail safe N

**Strategy for Data Synthesis:**

Effect sizes for both CNS (focusing on brain structural, neurophysiological, and neurochemical parameters) and non-CNS dysfunction (focusing on immune, cardiometabolic, and hypothalamic-pituitary-adrenal (HPA) parameters) in first episode psychosis (FEP) will be calculated using random effects meta-analyses.

Statistical comparison of summary effect size magnitudes between the 3 CNS systems (brain structural, neurochemical, and neurophysiological) and 3 non-CNS systems (immune, cardiometabolic, and HPA) will be performed using a Wald-type test.

**References**

1. Expert Panel on Detection E, Treatment of High Blood Cholesterol in A. Executive Summary of The Third Report of The National Cholesterol Education Program (NCEP) Expert Panel on Detection, Evaluation, And Treatment of High Blood Cholesterol In Adults (Adult Treatment Panel III). *JAMA* 2001; **285**(19): 2486-97.

2. Grundy SM, Cleeman JI, Daniels SR, et al. Diagnosis and management of the metabolic syndrome: an American Heart Association/National Heart, Lung, and Blood Institute Scientific Statement. *Circulation* 2005; **112**(17): 2735-52.

3. Alberti KG, Zimmet P, Shaw J, Group IDFETFC. The metabolic syndrome--a new worldwide definition. *Lancet* 2005; **366**(9491): 1059-62.

4. World Health Organization. Definition, Diagnosis and Classification of Diabetes Mellitus and its Complications: Report of a WHO Consultation. Part 1: Diagnosis and Classification of Diabetes Mellitus. Geneva, World Health Org., 1999.

5. M DEH, Correll CU, Bobes J, et al. Physical illness in patients with severe mental disorders. I. Prevalence, impact of medications and disparities in health care. *World Psychiatry* 2011; **10**(1): 52-77.

6. Harris MI, Klein R, Welborn TA, Knuiman MW. Onset of NIDDM occurs at least 4-7 yr before clinical diagnosis. *Diabetes Care* 1992; **15**(7): 815-9.

7. Danesh J, Whincup P, Walker M, et al. Low grade inflammation and coronary heart disease: prospective study and updated meta-analyses. *BMJ* 2000; **321**(7255): 199-204.

8. Keshavan MS, Tandon R, Boutros NN, Nasrallah HA. Schizophrenia, "just the facts": what we know in 2008 Part 3: neurobiology. *Schizophr Res* 2008; **106**(2-3): 89-107.

9. Moher D, Liberati A, Tetzlaff J, Altman DG, Grp P. Preferred Reporting Items for Systematic Reviews and Meta-Analyses: The PRISMA Statement. *Journal of Clinical Epidemiology* 2009; **62**(10): 1006-12.

10. Aromataris E, Fernandez R, Godfrey CM, Holly C, Khalil H, Tungpunkom P. Summarizing systematic reviews: methodological development, conduct and reporting of an umbrella review approach. *Int J Evid Based Healthc* 2015; **13**(3): 132-40.

11. Smith V, Devane D, Begley CM, Clarke M. Methodology in conducting a systematic review of systematic reviews of healthcare interventions. *BMC Med Res Methodol* 2011; **11**(1): 15.

12. Breitborde NJ, Srihari VH, Woods SW. Review of the operational definition for first-episode psychosis. *Early Interv Psychiatry* 2009; **3**(4): 259-65.

13. Sharif MO, Janjua-Sharif FN, Ali H, Ahmed F. Systematic reviews explained: AMSTAR-how to tell the good from the bad and the ugly. *Oral Health Dent Manag* 2013; **12**(1): 9-16.

14. Higgins JP, Thompson SG, Deeks JJ, Altman DG. Measuring inconsistency in meta-analyses. *BMJ* 2003; **327**(7414): 557-60.

15. Pillinger T, Beck K, Gobjila C, Donocik JG, Jauhar S, Howes OD. Impaired glucose homeostasis in first-episode schizophrenia: A systematic review and meta-analysis. *JAMA Psychiatry* 2017; **74**(3): 261-9.

16. Perry BI, McIntosh G, Weich S, Singh S, Rees K. The association between first-episode psychosis and abnormal glycaemic control: systematic review and meta-analysis. *Lancet Psychiatry* 2016.

17. Greenhalgh AM, Gonzalez-Blanco L, Garcia-Rizo C, et al. Meta-analysis of glucose tolerance, insulin, and insulin resistance in antipsychotic-naive patients with nonaffective psychosis. *Schizophr Res* 2016.

18. Misiak B, Stanczykiewicz B, Laczmanski L, Frydecka D. Lipid profile disturbances in antipsychotic-naive patients with first-episode non-affective psychosis: A systematic review and meta-analysis. *Schizophr Res* 2017.

19. Pillinger T, Beck K, Stubbs B, Howes O. Decreased total and LDL cholesterol but raised triglycerides in first-episode schizophrenia: a systematic review and meta-analysis. *In submission* 2017.

20. Flatow J, Buckley P, Miller BJ. Meta-analysis of oxidative stress in schizophrenia. *Biol Psychiat* 2013; **74**(6): 400-9.

21. Upthegrove R, Manzanares-Teson N, Barnes NM. Cytokine function in medication-naive first episode psychosis: a systematic review and meta-analysis. *Schizophr Res* 2014; **155**(1-3): 101-8.

22. Goldsmith DR, Rapaport MH, Miller BJ. A meta-analysis of blood cytokine network alterations in psychiatric patients: comparisons between schizophrenia, bipolar disorder and depression. *Mol Psychiatry* 2016.

23. Miller BJ, Buckley P, Seabolt W, Mellor A, Kirkpatrick B. Meta-analysis of cytokine alterations in schizophrenia: clinical status and antipsychotic effects. *Biol Psychiatry* 2011; **70**(7): 663-71.

24. Fernandes BS, Steiner J, Bernstein HG, et al. C-reactive protein is increased in schizophrenia but is not altered by antipsychotics: meta-analysis and implications. *Mol Psychiatry* 2016; **21**(4): 554-64.

25. Miller BJ, Gassama B, Sebastian D, Buckley P, Mellor A. Meta-analysis of lymphocytes in schizophrenia: clinical status and antipsychotic effects. *Biol Psychiatry* 2013; **73**(10): 993-9.

26. Berger M, Kraeuter AK, Romanik D, Malouf P, Amminger GP, Sarnyai Z. Cortisol awakening response in patients with psychosis: Systematic review and meta-analysis. *Neurosci Biobehav Rev* 2016; **68**: 157-66.

27. Chaumette B, Kebir O, Mam-Lam-Fook C, et al. Salivary cortisol in early psychosis: New findings and meta-analysis. *Psychoneuroendocrinology* 2016; **63**: 262-70.

28. Girshkin L, Matheson SL, Shepherd AM, Green MJ. Morning cortisol levels in schizophrenia and bipolar disorder: a meta-analysis. *Psychoneuroendocrinology* 2014; **49**: 187-206.

29. Gonzalez-Blanco L, Greenhalgh AM, Garcia-Rizo C, Fernandez-Egea E, Miller BJ, Kirkpatrick B. Prolactin concentrations in antipsychotic-naive patients with schizophrenia and related disorders: A meta-analysis. *Schizophr Res* 2016; **174**(1-3): 156-60.

30. Adriano F, Caltagirone C, Spalletta G. Hippocampal volume reduction in first-episode and chronic schizophrenia: A review and meta-analysis. *Neuroscientist* 2012; **18**(2): 180-200.

31. Walter A, Suenderhauf C, Harrisberger F, et al. Hippocampal volume in subjects at clinical high-risk for psychosis: A systematic review and meta-analysis. *Neuroscience and Biobehavioral Reviews* 2016; **71**: 680-90.

32. Adriano F, Spoletini I, Caltagirone C, Spalletta G. Updated meta-analyses reveal thalamus volume reduction in patients with first-episode and chronic schizophrenia. *Schizophrenia Research* 2010; **123**(1): 1-14.

33. Haijma SV, Van Haren N, Cahn W, Koolschijn PC, Hulshoff Pol HE, Kahn RS. Brain volumes in schizophrenia: a meta-analysis in over 18 000 subjects. *Schizophr Bull* 2013; **39**(5): 1129-38.

34. Vita A, de Peri L. Hippocampal and amygdala volume reductions in first-episode schizophrenia. *The British Journal of Psychiatry* 2007; **190**(3): 271.

35. de Peri L, Crescini A, Deste G, Fusar-Poli P, Sacchetti E, Vita A. Brain structural abnormalities at the onset of schizophrenia and bipolar disorder: A meta-analysis of controlled magnetic resonance imaging studies. *Current Pharmaceutical Design* 2012; **18**(4): 486-94.

36. Vita A, De Peri L, Silenzi C, Dieci M. Brain morphology in first-episode schizophrenia: A meta-analysis of quantitative magnetic resonance imaging studies. *Schizophrenia Research* 2006; **82**(1): 75-88.

37. Fusar-Poli P, Radua J, McGuire P, Borgwardt S. Neuroanatomical maps of psychosis onset: Voxel-wise meta-analysis of antipsychotic-naive vbm studies. *Schizophrenia Bull* 2012; **38**(6): 1297-307.

38. Erickson MA, Ruffle A, Gold JM. A Meta-Analysis of Mismatch Negativity in Schizophrenia: From Clinical Risk to Disease Specificity and Progression. *Biol Psychiat* 2016; **79**(12): 980-7.

39. Qiu YQ, Tang YX, Chan RC, Sun XY, He J. P300 aberration in first-episode schizophrenia patients: a meta-analysis. *PLoS One* 2014; **9**(6): e97794.

40. Chen KC, Lee IH, Yang YK, et al. P300 waveform and dopamine transporter availability: a controlled EEG and SPECT study in medication-naive patients with schizophrenia and a meta-analysis. *Psychological medicine* 2014; **44**(10): 2151-62.

41. Haigh SM, Coffman BA, Salisbury DF. Mismatch Negativity in First-Episode Schizophrenia: A Meta-Analysis. *Clinical EEG and neuroscience* 2017; **48**(1): 3-10.

42. Brugger S, Davis JM, Leucht S, Stone JM. Proton magnetic resonance spectroscopy and illness stage in schizophrenia--a systematic review and meta-analysis. *Biol Psychiatry* 2011; **69**(5): 495-503.
